# A Highly Asymmetric Gold(III) η^3^‐Allyl Complex

**DOI:** 10.1002/anie.201912315

**Published:** 2019-12-12

**Authors:** Marte Sofie Martinsen Holmsen, Ainara Nova, Sigurd Øien‐Ødegaard, Richard H. Heyn, Mats Tilset

**Affiliations:** ^1^ Department of Chemistry University of Oslo P.O. Box 1033 Blindern 0315 Oslo Norway; ^2^ Hylleraas Centre for Quantum Molecular Sciences Department of Chemistry University of Oslo P.O. Box 1033 Blindern 0315 Oslo Norway; ^3^ Department of Chemistry UiT-The Arctic University of Norway 9037 Tromsø Norway; ^4^ SINTEF Industry P.O. Box 124 Blindern 0314 Oslo Norway

**Keywords:** allyl ligands, gold(III) complexes, π complexes, structural elucidation

## Abstract

A highly asymmetric Au^III^ η^3^‐allyl complex has been generated by treating Au(η^1^‐allyl)Br(tpy) (tpy=2‐(*p*‐tolyl)pyridine) with AgNTf_2_. The resulting η^3^‐allyl complex has been characterized by NMR spectroscopy and X‐ray crystallography. DFT calculations and variable temperature ^1^H NMR suggest that the allyl ligand is highly fluxional.

## Graphical abstract

**Gold and dynamic**: The π coordination of an allyl group at the cyclometalated (N,C coordinated) Au^III^ moiety induces high asymmetry in the allyl group. DFT calculations and variable temperature ^1^H NMR spectroscopy suggest that the allyl ligand is highly fluxional.

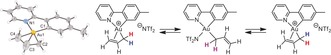

Transition‐metal allyl complexes have been thoroughly studied and are key intermediates in a variety of metal‐catalysed organic reactions, such as the widely used Pd‐catalysed Tsuji–Trost reaction which in one step gives access to highly functional compounds via nucleophilic addition to the η^3^ allyl in a regio‐ and stereospecific manner.^1^, ^2^ Despite that the allyl ligand is one of the classical unsaturated, delocalized ligands in organometallic chemistry, Au^III^ η^3^‐allyl complexes have been rarely described in the literature. There are a couple of reports on DFT calculations of such complexes and one experimental study in the gas phase using mass spectrometry techniques.^3^, ^4^ A few Au^III^ η^1^ allyl complexes^5^ have been reported together with a handful of Au^I^ η^1^‐allyl complexes.^6^ Herein, we report for the first time the generation and full characterization of an isolable Au^III^ η^3^‐allyl complex.

Treatment of Au(OAc^F^)_2_(tpy) (**1**; OAc^F^=OCOCF_3_) with allylmagnesium bromide according to our previously developed methodology^7^ led to the formation of Au(η^1^‐allyl)Br(tpy) (**2**), with the allyl group *trans* to tpy‐*N* (Scheme 1, left). Complex **2** was obtained in 52–69 % yield and characterized by NMR, MS, elemental analysis and X‐ray diffraction analysis.^13^ The characteristic resonances of the protons on the allyl ligand are observed in the ^1^H NMR spectrum of **2**; the three vinylic signals are found at *δ*=6.28 (H^b^, see labelling in Scheme 1), 5.48 (H^c^), and 5.02 (H^d^). The two allylic hydrogens H^a^ are chemically equivalent and give rise to one resonance at *δ* 3.39. A ^1^H–^1^H NOESY experiment established that the η^1^ allyl ligand is located *trans* to tpy‐N; a NOE is observed between H^6′^ and H^a^, H^b^, and H^c^ (Figure 1).

**Figure 1:**
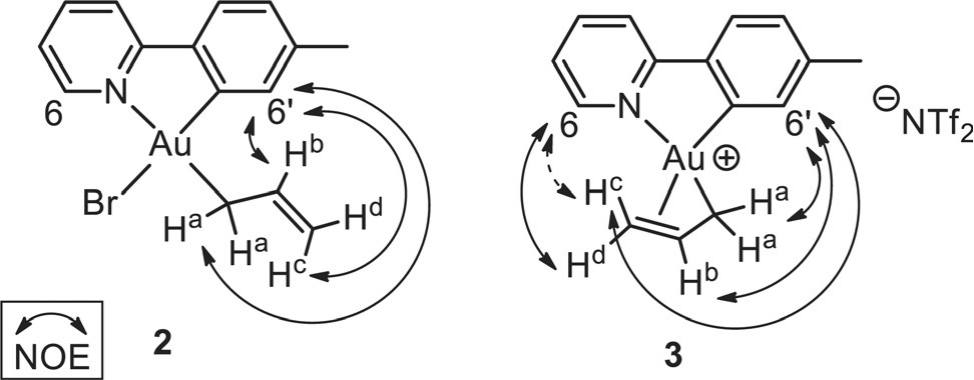
Depiction of selected ^1^H–^1^H NOE correlations of complexes **2** and **3**.

**Scheme 1:**
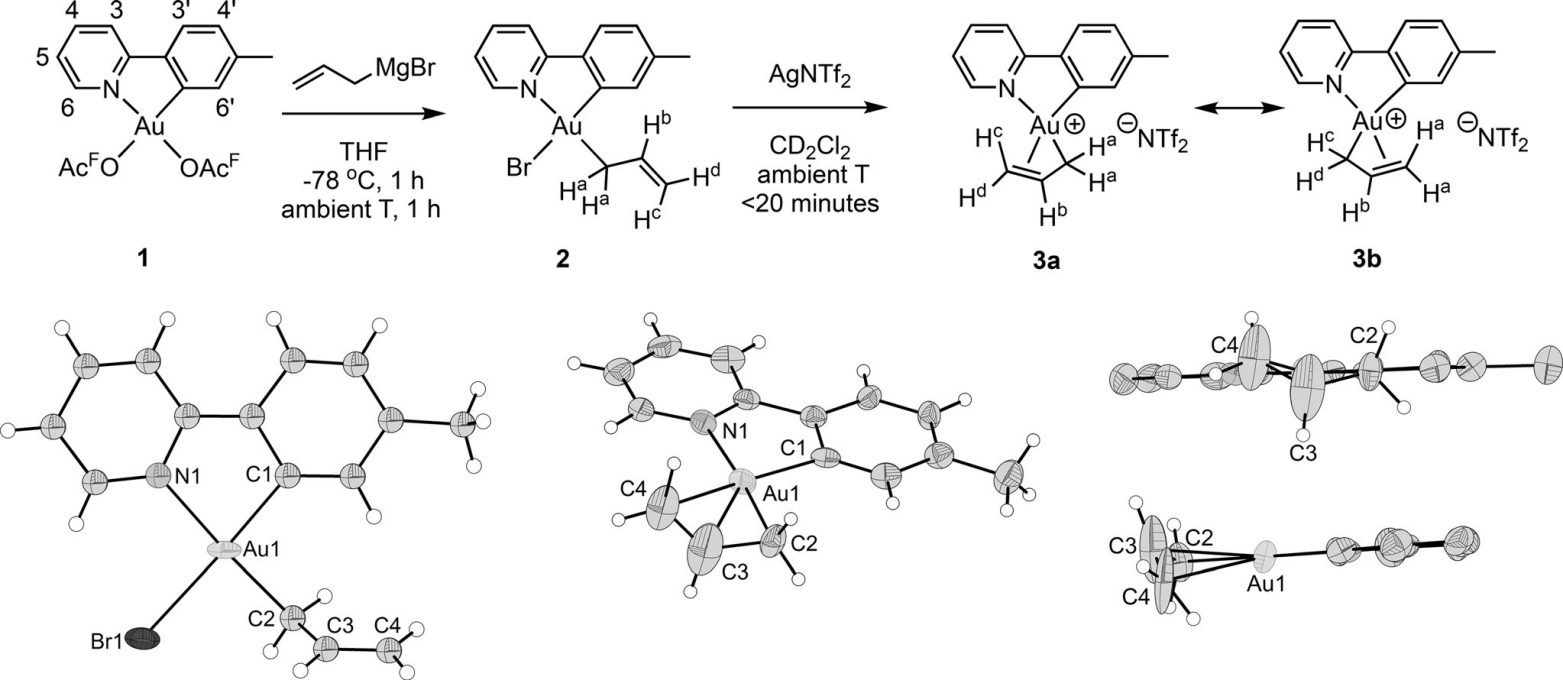
Top: Generation of Au^III^ η^1^‐ and η^3^‐allyl complexes **2** and **3**. Bottom: Crystallographic structure determination of **2** (left).^13^ Owing to twinning and disorder limiting the high‐resolution diffraction in the measured crystal, only Au and Br are refined as thermal ellipsoids (set at 50 % probability). ORTEP plot of the cationic part of complex **3** with thermal ellipsoids set at 50 % probability (right). Selected bond lengths [Å] and bond angles [°] for **2**: Au1–N1 2.11(3), Au1‐C1, 2.02(4), Au1–C2 2.10(4), Au1–Br1 2.493(5), C2–C3 1.41(5), C3–C4 1.31(5); Br1‐Au1‐N1 95.0(9), N1‐Au1‐C1 81.7(15), C1‐Au1‐C2 94.9(16), C2‐Au1‐Br1 88.7(12). Selected bond lengths [Å] and bond angles [°] for **3**: Au1–N1 2.119(16), Au1–C1 2.04(2), Au1–C2 2.062(19), Au1–C3 2.21(2), Au1–C4 2.35(2), C2–C3 1.43(3), C3–C4 1.22(4); C4‐Au1‐N1 109.8(8), N1‐Au1‐C1 81.0(7), C1‐Au1‐C2 104.1(8), C2‐Au1‐C4 65.2(8). Selected DFT optimized bond lengths [Å] and bond angles [°] for **3**: Au1–N1 2.099, Au1–C1 2.031, Au1–C2 2.090, Au1–C3 2.224, Au1–C4 2.329, C2–C3 1.438, C3–C4 1.382; C4‐Au1‐N1 107.86, N1‐Au1‐C1 80.62, C1‐Au1‐C2 104.51, C2‐Au1‐C4 66.67, C1‐Au1‐C4 168.87, N1‐Au1‐C2 174.06, C2‐C3‐C4 119.45.

Addition of AgNTf_2_ to a CD_2_Cl_2_ solution of **2** led to the formation of Au(η^3^‐allyl)(tpy) (**3**) as the major product (Scheme 1, right), together with traces of what appeared to be a decomposition product. Complex **3** was characterized by NMR and X‐ray diffraction analysis.^13^ A comparison of the ^1^H NMR spectra of **3** and **2** (Table 1 and Supporting Information) shows that H^b^ and H^d^ are found at higher chemical shift in **3** compared to in **2** (Δ*δ*=0.22 (H^b^) and 0.66 (H^d^)), whereas H^c^ is observed at a lower δ (Δ*δ*=−0.18). The two H^a^ are found at a higher chemical shift (Δ*δ*=0.41).

**Table 1:** δ(^1^H) and ^*1*^ *J*(^1^H‐^13^C) data for the allylic groups of complexes **2** ^[a]^ and **3** ^[b]^ in CD_2_Cl_2_.

Atom	**2**	^*1*^ *J* (^1^H‐^13^C)	**3** (Δδ)	^*1*^ *J* (^1^H‐^13^C)
H^a^	*δ*=3.39	142 Hz	*δ*=3.80 (+0.41)	156 Hz
H^b^	*δ*=6.28	154 Hz	*δ*=6.50 (+0.22)	164 Hz
H^c^	*δ*=5.48	154 Hz	*δ*=5.30 (−0.18)	158 Hz
H^d^	*δ*=5.02	158 Hz	*δ*=5.68 (+0.66)	165 Hz

[a] Measured at 600 MHz (ca. 27 °C). [b] Measured at 800 MHz (ca. 28 °C). Δ*δ*=δ(**3**)−δ(**2**). Coupling constants were measured from a non‐decoupled ^1^H–^13^C HMBC experiment.

Complex **3** can be described by the two Lewis (resonance) structures **3 a** and **3 b** (Scheme 1). The ^1^H NMR data, however, suggest the prevalence of one structure over the other; three protons are observed in the vinylic region (H^b^, H^c^, and H^d^; see Table 1) and the two H^a^ are found at a significantly lower ppm value. This is rather unusual for η^3^ allyl complexes; normally the *anti* protons (defined relative to the central proton which is usually found at around *δ*=6.5; H^b^ in complex **3**) are found at *δ*=1–3, whereas the *syn* protons are found at larger ppm values, around *δ*=2–5.^2^

Furthermore, there is only a small increase (by 14 Hz) in ^*1*^ *J*(H^a^‐C2) going from **2** to **3** (Table 1) indicating that the sp^3^ hybridization of C2 remains essentially unchanged. This result also agrees with the thermodynamic preference of having the high *trans* influence C(sp^3^) end of the allyl ligand *trans* to the lower *trans* influence ligand tpy‐N, instead of the higher *trans* influence tpy‐C, and leads us to infer the structural preference of **3 a** over **3 b**. In symmetric η^3^‐allyl complexes the *syn* and *anti* H^a^ protons usually give rise to two distinct signals. However, if double bond decoordination^8^ followed by rotation around the MCH_2_−CHCH_2_ bond and re‐coordination occurs relatively fast on the NMR time scale, the resonances for these two protons will coalesce into one averaged resonance. The fact that a coalesced signal is seen for the two H^a^, but not for H^c^ and H^d^, suggests that double bond decoordination/recoordination of the η^3^‐allyl ligand occurs selectively *trans* to the tpy‐C atom in **3 a**. No evidence is seen in the NMR spectra for an analogous process starting from Lewis structure **3 b** which would lead to a coalescence of the resonances of H^c^ and H^d^. This supports the notion that resonance structure **3 b** is a minor contributor due to the unfavourable *trans* relationship between the C(sp^3^) end of the allyl group and the coordinating tpy‐C atom.

The structure and dynamic behaviour of **3** were explored by DFT calculations at the PBE0 level, including solvation by dichloromethane (see Supporting Information for computational details). The optimized structure shows inequivalent C−C bonds in the allyl ligand of 1.438 Å and 1.382 Å for C2–C3 and C3–C4, respectively, in agreement with **3 a** as the predominant Lewis structure (Scheme 1). Double bond decoordination to furnish an η^1^‐allyl species occurred favourably only *trans* to the coordinating tpy‐C atom and led to two structures with the empty coordination site *trans* to the tpy‐C atom and the η^1^‐allyl *trans* to the tpy‐N atom (**4**, 12.3 kcal mol^−1^; and **5**, 11.9 kcal mol^−1^, Scheme 2). Interestingly, two different TSs of similar energies (**TS3‐4** and **TS5‐3′**) were located connecting these two η^1^ allylic intermediates with enantiomers **3** and **3′**, indicating the existence of two TSs for the double bond decoordination. Starting from a given enantiomer, these TSs correspond to clockwise and counter‐clockwise rotations of the Au−C bond (see ESI). The two η^1^‐allyl intermediates **4** and **5** are also connected by a TS involving rotation of the σ(C2–C3) bond (**TS4‐5**). The energy associated with this TS (17.7 kcal mol^−1^) is the highest in the computed energy landscape that facilitates the exchange of H^a^ and H^a′^, with barriers that are consistent with a process that occurs at room temperature.

**Scheme 2:**
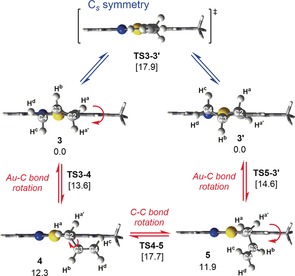
Double bond decoordination and subsequent rotation and recoordination in complex **3** as shown will cause an averaging of the resonances of the two H^a^ into one signal. Optimized geometries (PBE0‐D3, SDD/6–311+G**, SMD=dichloromethane) and Δ*G* energies (kcal mol^−1^) for all intermediates and TSs involved in the equilibria between **3** and its enantiomer. Red=chiral pathway, blue=symmetric pathway. See text for details.

The process described in Scheme 2 (red pathway) involves the interconversion of one enantiomer of **3** to its enantiomeric counterpart (**3′**) via a chiral pathway. It has been argued^9^ that such a process is not in violation of the principle of microscopic reversibility provided that there exists a degenerate alternative pathway, of opposite chirality but energetically degenerate to the first one (see Supporting Information). Burkey and co‐workers^10^ recently reported metallacycle ring inversions that were suggested to occur by chiral, degenerate pathways. Interestingly, the interconversion of the enantiomers **3** and **3′** by this pathway does not involve a *C*_s_ symmetric intermediate or transition state which might be considered to arise from double bond decoordination and Au−C and C−C bond rotations. Optimization of the η^1^‐allyl geometry within *C*_s_ symmetry constraints leads to a *C*_s_ symmetric TS at 17.9 kcal mol^−1^ (blue pathway, Scheme 2). This transition state was found to directly connect **3** and **3′**. The similar energies obtained for the symmetric pathway and the chiral one (Scheme 2) suggest the co‐existence of the two pathways at the experimental conditions.

Complex **3** slowly decomposes at ambient temperature and complete NMR characterization was therefore performed at 7 °C. The resonances of H^a^, H^d^, and H^6^, as well as several of the ^13^C NMR resonances are broadened at this temperature (see Supporting Information). The temperature‐dependent broadening phenomena in the ^1^H and ^13^C NMR spectra further support the dynamic behaviour of the allyl ligand on the NMR time scale. Selected key ^1^H–^1^H NOE correlations in complex **3** are depicted in Figure 1. A NOE between H^d^ and H^6^ is observed, which is not observed in complex **2**, indicating a coordination of the double bond to Au, *trans* to the tpy‐C atom. In contrast, H^c^ (bonded to the same C as H^d^) shows a NOE with H^6′^, but upon increasing the intensity of the peaks in the NOESY spectrum, what appears to be a weak NOE between H^c^ and H^6^ becomes visible. These observations might indicate that **3**, with the allyl ligand bound in an η^3^ fashion, interconverts to the corresponding η^1^‐allyl complex during the time scale of the NMR experiment, as depicted in Scheme 2.

Assuming the behaviour depicted in Scheme 2, a further slowing of the process by lowering the temperature will cause the resonance of the two H^a^ to split into two signals. Thus, decreasing the temperature to −42.3 °C led to significant broadening of the resonances of H^a^, H^c^, and H^d^ in the ^1^H NMR spectrum of **3** (see Figure 2). At this point, the signals of H^6^ (see Supporting Information) and H^b^ are also broadened, but to a lesser extent. Interestingly, upon lowering the temperature further, the resonance of H^a^ undergoes de‐coalescence and eventually emerges as three resonances. At −55.5 °C these are significantly broadened and are barely discernible as three featureless, broadened distortions of the baseline. At −79.2 °C these resonances, at *δ*=4.26, 3.82, and 3.09, are sharper and integrate for approximately 1H, 1H, and 2H, respectively (see the spectrum at the bottom of Figure 2 and Supporting Information). At this temperature, two resonances are also observed for H^d^ (each integrating for ca. 1H), whereas the signals of H^b^ and H^c^ each appear as one broadened resonance (ca. 2H each). Furthermore, two sets of peaks for most of the resonances of the tpy ligand are observed (see Supporting Information). The broadening/coalescence behaviour is reversible, as evidenced by the restoration of signals upon sample heating. Based on these observations it is suggested that there is an interconversion between the η^3^‐allyl complexes **3** and **3′**, and the η^1^‐allyl complexes **4** and **5** (perhaps with NTf_2_ or solvent coordinated *trans* to the tpy‐C atom) in solution (Scheme 2). At −79.2 °C, this process is slow enough to enable the detection of coexisting η^3^ (**3**/**3′**) and η^1^ (**4**/**5**, with an eventual coordinated counteranion or solvent molecule) forms by ^1^H NMR spectroscopy. In an η^1^‐allyl complex, the two H^a^ are chemically equivalent, and therefore it is suggested that the resonance at *δ*=3.09 arises from such a complex; this chemical shift is slightly lower than that observed for the two chemically equivalent H^a^ in η^1^‐allyl complex **2** (*δ*=3.39) and nearly the same as that in [Au(η^1^‐allyl)(CD_3_CN)(tpy)]^+^[NTf_2_]^−^ (*δ*=3.12, see Supporting Information). The resonances at *δ*=4.26 and 3.82 are thus assigned to η^3^‐allyl complex **3**. Based on the findings from low‐temperature NMR spectroscopy, what is observed by ^1^H NMR spectroscopy at room temperature is not strictly an η^3^‐allyl complex, but rather the averaged signals arising from the η^3^‐η^1^‐η^3^ interconversions whereby complex **3** interconverts to and equilibrates with an η^1^‐allyl complex.

**Figure 2:**
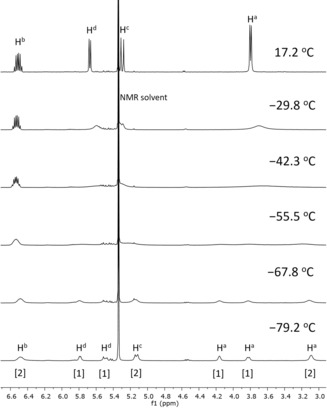
Variable temperature ^1^H NMR (500 MHz, CD_2_Cl_2_) spectra of the allylic region of **3** (for full spectra, see Supporting Information). Numbers in [brackets] under the bottom spectrum show the approximate relative integrals of the signals of interest.

The DFT free energies obtained for intermediates **4** and **5** do not account for the existence of η^1^ allyl intermediates in solution. However, upon coordination of NTf_2_ at Au (**4‐NTf_2_** and **5‐NTF_2_**, see Supporting Information), these species became almost isoenergetic to the η^3^‐allyl complex **3** (Δ*G*=−3.6 kcal mol^−1^).^11^ Therefore, the equilibrium observed in solution may involve coordination and decoordination of NTf_2_ (see Figure S33).

Crystallographic structure determination of complexes **2** and **3** were performed and selected parameters are given in Scheme 1. As can be seen from Scheme 1, complex **2** is an η^1^ allyl complex with the allyl *trans* to the tpy‐N atom and Br *trans* to the coordinating tpy‐C atom, in full agreement with the NMR data. In complex **3**, the double bond of the allyl has coordinated *trans* to tpy‐C to form an η^3^‐allyl complex as depicted in Scheme 1.

In **3**, the C(sp^3^) end of the allyl ligand (C2) is more tightly bound to Au than the C(sp^2^) C=C carbon atoms (C3 and C4) with Au−C bonds of 2.062(19), 2.21(2) and 2.35(2) Å, respectively, indicative of a highly asymmetric allyl complex which is best described by Lewis structure **3 a** and not **3 b**.

The allyl ligand in **3** is more asymmetrically bonded than what is seen in related Pd^II^(*N*,*C*) cyclometalated complexes reported previously^12^ (where the chelate *N* is a pyridine‐N atom, and the chelate *C* is either an aryl‐C or a NHC‐C atom), with Pd–allyl bonds of 2.105(5)/2.095(4) Å (Pd–C2), 2.135(5)/2.152(4) Å (Pd–C3) and 2.257(5)/2.222(5) Å (Pd–C4). In addition, in complex **3**, the DFT determined C2–C3 distance is significantly longer than C3–C4 (1.438 vs. 1.382 Å), again indicating an asymmetric allyl complex. The distances are taken from DFT calculations because the experimental C−C bond lengths of the allyl ligand have a high uncertainty due to the absence of high resolution diffraction signals, probably originating from disorder and twinning in the crystals. However, the differences in crystallographically determined bond lengths are still significant. DFT calculations were also used to determine the geometry expected for the isoelectronic, neutral complex Pt(η^3^‐allyl)(tpy) (Figure S34). In this case, key bond lengths were found to be 1.433 Å for C2–C3, 1.402 Å for C3–C4, 2.091 Å for Pt–C2 and 2.221 Å for Pt–C4. While this system is also highly asymmetric, the differences between the C−C and M−C bond lengths are larger for Au^III^ (0.056 and 0.239 Å, respectively, for M=Au; 0.031 and 0.130 Å, respectively, for M=Pt).

In conclusion, we have generated and fully characterized the first Au^III^ η^3^‐allyl complex.^14^ NMR spectroscopy and XRD analysis together with DFT calculations show that the allyl ligand bound to Au is highly asymmetric. This asymmetric bonding appears to be dictated by the different *trans* influence of the coordinating atoms of the ancillary ligands (tpy‐N vs. tpy‐C). We are currently investigating how this asymmetry will affect the reactivity of this class of complexes.

## Conflict of interest

The authors declare no conflict of interest.

## Supporting information

As a service to our authors and readers, this journal provides supporting information supplied by the authors. Such materials are peer reviewed and may be re‐organized for online delivery, but are not copy‐edited or typeset. Technical support issues arising from supporting information (other than missing files) should be addressed to the authors.

Supplementary
