# Supplementary material for: A Highly Asymmetric Gold(III) η3‐Allyl Complex
Source: Angew Chem Int Ed Engl. 2019 Dec 12;59(4):1516–20. doi: 10.1002/anie.201912315 (PMC7003771; doi:10.1002/anie.201912315)
Supplement: Supplementary file 1 — Supplementary [file ANIE-59-1516-s001.pdf]

Angewandte Chemie
Eine Zeitschrift der Gesellschaft Deutscher Chemiker

Supporting Information

A Highly Asymmetric Gold(III) η^3-Allyl Complex

Marte Sofie Martinsen Holmsen, Ainara Nova, Sigurd Øien-Ødegaard, Richard H. Heyn, and Mats Tilset**

anie_201912315_sm_miscellaneous_information.pdf

Electronic Supplementary Information

Table of contents

p. S2 Experimental section – general procedures
p. S3 Preparation and characterization data complex **2**
p. S4 Preparation and characterization data for complex **3**
p. S5 Preparation and characterization data for [Au(η^1-allyl)(CD_3CN)(tpy)]$^+$[NTf_2]$^-$
p. S6 NMR spectra of complex **2**
p. S11 NMR spectra of complex **3**
p. S18 NMR spectra of [Au(η^1-allyl)(CD_3CN)(tpy)]$^+$[NTf_2]$^-$
p. S23 Variable temperature ^{1}H NMR of complex **3**
p. S26 Crystallographic structure determination of complexes **2** and **3**
p. S32 Reaction pathways for the interconversion of the η^3-allyl enantiomers **3** and **3'**
p. S32 Reaction pathway for the interconversion of the η^1-allyl intermediates **4** and **5**
p. S33 M(η^3-allyl)(tpy) with M=Pt(II) and Au(III)
p. S33 Computational details
p. S34 Optimized coordinates and energies
p. S43 References

General procedures

Complex **1** was synthesized as reported previously.[1] CD_2Cl_2 was dried over molecular sieves prior to use. CH_2Cl_2 and THF were purified using a MB SPS-800 solvent purifying system from MBraun. All solvents (including NMR solvents) utilized inside the glove box were dried over molecular sieves and degassed by the freeze-pump-thaw method prior to use. Distilled water was used in the work up of complex **2**. All other reagents and solvents were used as received. An inert atmosphere argon-filled glove box of the type UNIlab Pro from MBraun was used. All glassware was dried prior to use and all reactions were performed under argon, except for the work up of complex **2**. As a precaution, all reactions were performed in the absence of light. NMR spectra were recorded on Bruker Avance AVI600, DRX500, DPX200 and AVIIIHD800. ^1H and ^{13}C NMR spectra have been referenced relative to the residual solvent signals (CD_2Cl_2: $\delta(^1H)$ 5.34, $\delta(^{13}C)$ 53.84; CD_3CN: $\delta(^1H)$ 1.94, $\delta(^{13}C)$ 1.32, 118.26). ^{19}F NMR has been referenced to $CFCl_3$ by using C_6F_6 (−164.9 ppm with respect to $CFCl_3$ at 0 ppm) as an external standard by adding a sealed capillary containing C_6F_6 to the NMR sample. The peaks in the ^1H NMR spectra were assigned by the aid of 2D NMR techniques such as COSY, HSQC, HMBC, and NOESY according to the numbering scheme shown in Figure 1. For the variable temperature ^1H NMR experiments, the temperature inside the probe was measured using a Delta OHM HD9214 thermometer fitted inside a NMR tube containing CD_2Cl_2. For the remaining NMR spectra, the exact temperature was not measured, and the temperature given might therefore deviate from the actual temperature of the sample. Mass spectra (ESI) were obtained on a Bruker maXis II ETD spectrometer. Elemental analysis was performed by Microanalytisches Laboratorium Kolbe, Mülheim an der Ruhr, Germany.

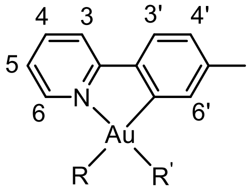

Figure 1. Numbering scheme used for reporting the NMR data.

Synthesis of complex 2

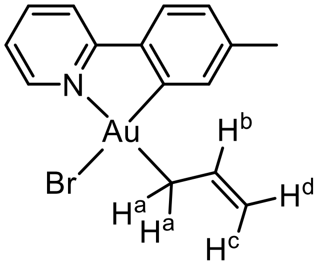

2

Complex **1** (200.0 mg, 0.3383 mmol, 1.0 equiv) was dissolved in THF and cooled down to *ca.* −78 °C (dry ice/acetone). Allyl magnesium bromide (0.50 mL 1.0 M solution in Et_2O, 0.50 mmol, 1.5 equiv) was added and the solution was stirred at *ca.* −78 °C in the absence of light for 1 h, followed by stirring at ambient temperature for 1 h. The volatiles were removed under reduced pressure and the remaining solid was dissolved in CH_2Cl_2 (50 mL). The solution was filtered and washed with distilled water (4 x 25 mL, pH ≈ 7). CH_2Cl_2 (100 mL) was added and the organic phase was dried over Na_2SO_4 and filtered. The solvent was removed under reduced pressure and the resulting solid was purified by flash chromatography (silica gel 60, CH_2Cl_2) furnishing complex **2** (99.7 mg, 0.205 mmol, 61%) as a white solid.

^{1}H NMR (800 MHz, CD_2Cl_2): δ 9.54 (ddd, 1H, J = 5.5, 1.5, 0.7 Hz, **H^6**), 7.99 (ddd, 1H, J = 8.0, 7.5, 1.6 Hz, **H^4**), 7.92 (d, 1H, J = 8.1 Hz, **H^3**), 7.69 (d, 1H, J = 7.8 Hz, **$H^{3'}$**), 7.52 (s, 1H, **$H^{6'}$**), 7.43 (ddd, 1H, J = 7.4, 5.6, 1.2 Hz, **H^5**), 7.23 (dq, 1H, J = 7.8, 0.7 Hz, **$H^{4'}$**), 6.28 (ddt, 1H, $^3J_{trans}$ = 17.0 Hz, $^3J_{cis}$ = 10.0 Hz, $^3J_{Ha\text{-}Hb}$ = 8.2 Hz, **H^b**), 5.48 (ddt, 1H, $^3J_{trans}$ = 17.0 Hz, $^2J_{gem}$ = 2.2 Hz, $^4J_{Ha\text{-}Hc}$ = 0.9 Hz, **H^c**), 5.02 (dd, 1H, $^3J_{cis}$ = 10.0 Hz, $^2J_{gem}$ = 2.2 Hz, **H^d**), 3.39 (d, 1H, $^3J_{Ha\text{-}Hb}$ = 8.1 Hz, **H^a**), 2.48 (s, 3H, ArC**H_3**).

^{13}C NMR (201 MHz, CD_2Cl_2): δ 161.9 (Ar**C**), 149.5 (Ar**C**), 149.3 (Ar**C**), 142.6 (Ar**C**), 141.5 (Ar**C**), 141.0 (Ar**C**), 139.0 (**C**H^b), 130.7 (Ar**C**), 128.8 (Ar**C**), 125.6 (Ar**C**), 124.2 (Ar**C**), 119.9 (Ar**C**), 114.9 (**C**$H_2^{c,d}$), 34.2 (**C**H_2^{a}), 22.1 (Ar**C**H_3). The resonance at δ 149.5 appear as a doublet due to insufficient ^{1}H decoupling.

MS (ESI, MeCN): *m/z* (rel. %): 508/510 ($[M+Na]^+$, 8/8), 406 ($[M-Br]^+$, 100), 360 (27).

HRMS (ESI, MeCN): Found: 507.9944; calcd. for $C_{15}H_{15}Au^{79}BrNNa$: 507.9946, Found: 406.0864; calcd. for $C_{15}H_{15}AuN$ 406.0865.

Elemental analysis: Anal. calcd. for $C_{15}H_{15}AuBrN$: C, 37.06; H, 3.11; N, 2.88. Found: C, 37.17; H, 3.10; N, 2.82.

Generation of complex 3

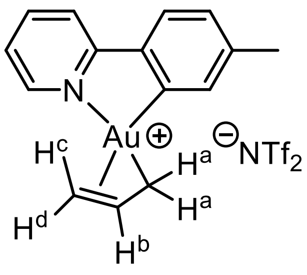

3

Complex **2** (5 mg, 0.01 mmol, 1 equiv) was dissolved in CD_2Cl_2 and added to $AgNTf_2$ (6 mg, 0.02 mmol, 2 equiv) inside a glove box. A white precipitate was formed immediately after the addition to $AgNTf_2$. The solution was transferred to a NMR tube and the NMR tube was taken out of the glove box. ^{1}H NMR *ca.* 20 minutes after mixing revealed that one major product (**3**) was formed together with small amounts of what is believed to be a decomposition product. Complex **3** has not been isolated and was only characterized in the CD_2Cl_2 solution in which it was generated. Complex **3** decomposes over time at ambient temperature; therefore the NMR characterization was performed at 7 °C.

^{1}H NMR (600 MHz, CD_2Cl_2, 27 °C): δ 8.84 (d, 1H, *J* = 5.2 Hz, **H^6**), 8.16 (ddd, 1H, *J* = 8.0, 7.8, 1.5 Hz, **H^4**), 8.03 (d, 1H, *J* = 8.2, **H^3**), 7.78 (d, 1H, *J* = 8.0 Hz, **$H^{3'}$**), 7.56 (s, 1H, **$H^{6'}$**), 7.53 (ddd, 1H, *J* = 7.5, 5.7, 1.2 Hz, **H^5**), 7.31 (dq, 1H, *J* = 7.9, 0.6 Hz, **$H^{4'}$**), 6.50 (ddt, 1H, $^3J_{trans}$ = 15.9 Hz, $^3J_{cis}$ = 8.9 Hz, $^3J_{\text{Ha-Hb}}$ = 8.9 Hz, **H^b**), 5.68 (d, 1H, $^3J_{cis}$ = 8.9 Hz, **H^d**), 5.30 (d, 1H, $^3J_{trans}$ = 15.8 Hz, **H^c**), 3.80 (d, 1H, $^3J_{Ha\text{-}Hb}$ = 8.9 Hz, **H^a**), 2.46 (s, 3H, ArC**H_3**).

^{1}H NMR (600 MHz, CD_2Cl_2, 7 °C): δ 8.85 (br. d, 1H, *J* = 5.3 Hz, **H^6**), 8.15 (ddd, 1H, *J* = 8.0, 7.7, 1.5 Hz, **H^4**), 8.02 (d, 1H, *J* = 8.2, **H^3**), 7.76 (d, 1H, *J* = 7.9 Hz, **$H^{3'}$**), 7.55 (s, 1H, **$H^{6'}$**), 7.53 (ddd, 1H, *J* = 7.5, 5.7, 1.2 Hz, **H^5**), 7.29 (d, 1H, *J* = 7.9 Hz, **$H^{4'}$**), 6.45 (ddt, 1H, $^3J_{trans}$ = 15.9 Hz, $^3J_{cis}$ = 8.9 Hz, $^3J_{\text{Ha-Hb}}$ = 8.9 Hz, **H^b**), 5.65 (br. d, 1H, $^3J_{cis}$ = 8.5 Hz, **H^d**), 5.29 (d, 1H, $^3J_{trans}$ = 15.8 Hz, **H^c**), 3.77 (br. d, 1H, $^3J_{Ha\text{-}Hb}$ = 8.2 Hz, **H^a**), 2.44 (s, 3H, ArC**H_3**).

^{19}F NMR (188 MHz, CD_2Cl_2, 25 °C): δ −76.3 (C**F_3**)

^{13}C NMR (151 MHz, CD_2Cl_2, 7 °C): δ 162.3 (br., Ar**C**), 153.1 (br., **C**H^6), *ca*. 151 (Ar**C**), 143.9 (Ar**C**), 142.8 (**C**H^4), 141.3 (Ar**C**), 135.5 (br., **C**$H^{6'}$), 134.6 (br., **C**H^b), 130.0 (**C**$H^{4'}$), 126.2 (**C**$H^{3'}$), 125.4 (**C**H^5), 121.0 (**C**H^3), 119.9 (q, *J* = 322.2 Hz, **C**F_3), 106.1 (br., **C**$H_2^{c,d}$), 49.8 (br., **C**H_2^a), 21.7 (Ar**C**H_3). The resonance at *ca.* δ 151 was broadened into the baseline, and was only observed indirectly in a ^{1}H-^{13}C HMBC experiment.

Generation of [Au(η^1-allyl)(CD_3CN)(tpy)]$^+$[NTf_2]$^-$

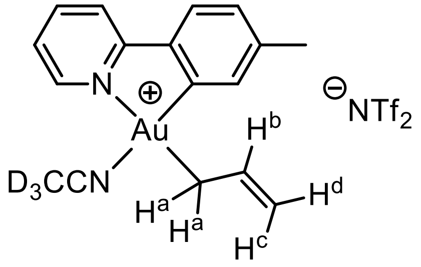

[Au(η^1-allyl)(CD_3CN)(tpy)]$^+$[NTf_2]$^-$

Complex **2** (5 mg, 0.01 mmol, 1 equiv) was dissolved in CD_3CN and added to $AgNTf_2$ (6 mg, 0.02 mmol, 2 equiv) inside a glove box. A white precipitate was formed immediately after the addition to $AgNTf_2$. The solution was transferred to a NMR tube and the NMR tube was taken out of the glove box. ^{1}H NMR *ca.* 20 minutes after mixing revealed that one product is formed which is, based on NMR characterization, assigned to be [Au(η^1-allyl)(CD_3CN)(tpy)]$^+$[NTf_2]$^-$. [Au(η^1-allyl)(CD_3CN)(tpy)]$^+$[NTf_2]$^-$ has not been isolated and was only characterized in the CD_3CN solution in which it was generated.

^{1}H NMR (600 MHz, CD_3CN): δ 8.71 (br. s, 1H, **H^6**), 8.16 (apparent triplet, 1H, *J* = 7.7 Hz, **H^4**), 8.08 (d, 1H, *J* = 7.7 Hz, **H^3**), 7.77 (d, 1H, 7.6 Hz, **H$^{3'}$**), 7.58 (apparent triplet, 1H, *J* = 6.3 Hz, **H^5**), 7.43 (s, 1H, **H$^{6'}$**), 7.27 (d, 1H, *J* = 7.7 Hz, **H$^{4'}$**), 6.24 (m, 1H, **H^b**), 5.53 (d, 1H, *J* =16.9 Hz, **H^c**), 5.18 (d, 1H, *J* = 9.9 Hz, **H^d**), 3.12 (d, 1H, *J* = 8.1 Hz, **H^a**), 2.43 (s, 1H, ArC**H$_3$**). All the resonances in the ^{1}H NMR spectrum are slightly broadened.

^{13}C NMR (151 MHz, CD_3CN): δ 160.4 (Ar**C**), 148.9 (**C**H^6), 142.8 (Ar**C**), 143.3 (**C**H^4), 142.2 (Ar**C**), 138.5 (Ar**C**), 138.1 (**C**H^b), 132.2 (**C**H$^{6'}$), 130.7 (**C**H$^{4'}$), 127.2 (**C**H$^{3'}$), 125.8 (**C**H^5), 121.6 (**C**H^3), 120.9 (q, *J* = 320.8 Hz, **C**F$_3$), 117.1 (**C**H$_2^{c,d}$), 38.7 (**C**H$_2^a$), 21.7 (Ar**C**H$_3$).

NMR spectra of complex 2

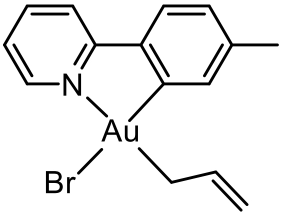

2

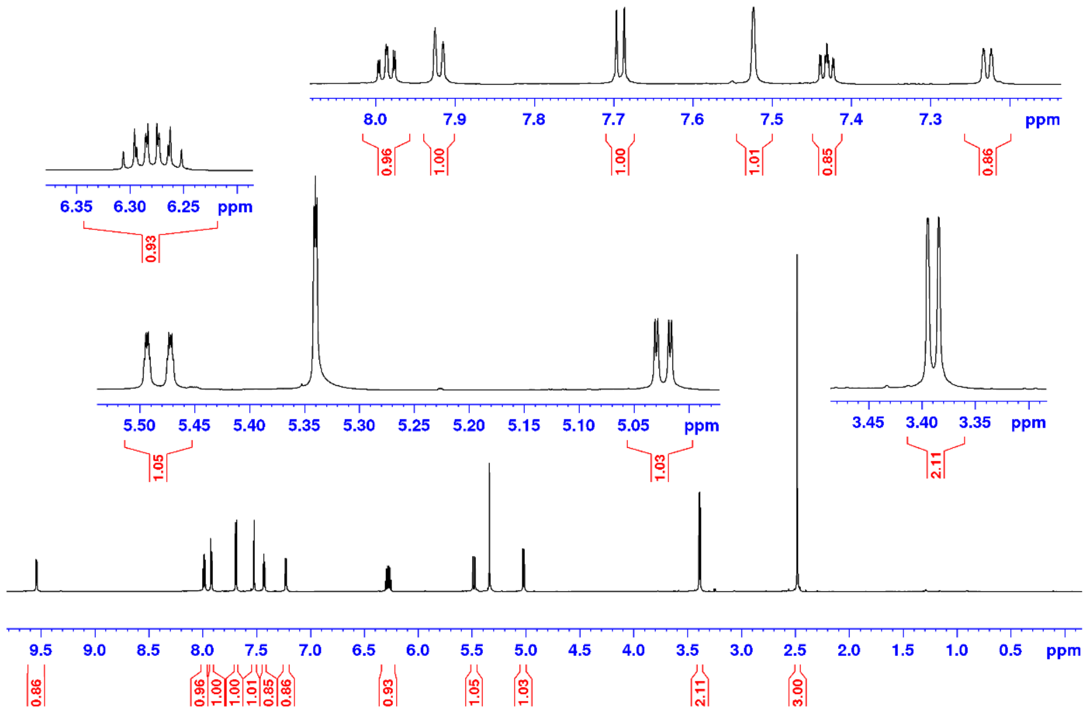

Figure S 1. ^{1}H NMR (800 MHz, CD_2Cl_2) spectrum of complex **2**.

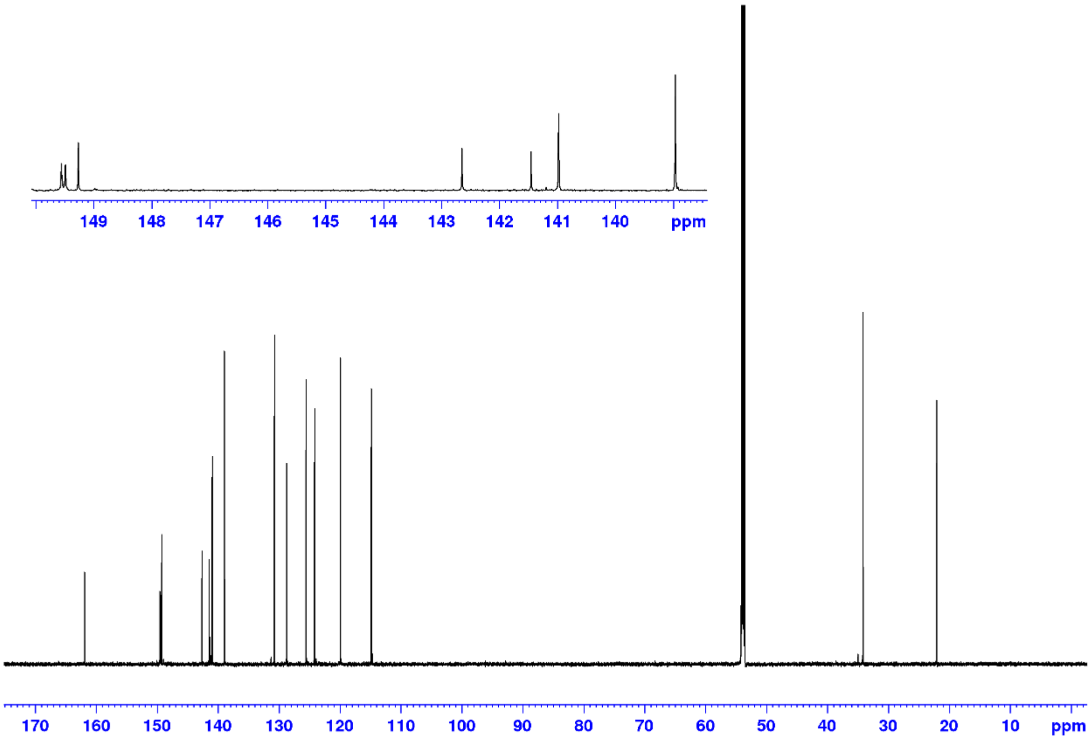

Figure S 2. ^{13}C NMR (201 MHz, CD_2Cl_2, ns = 4096, d1 = 3 s) spectrum of complex **2**. The resonance at δ 149.5 appears as a doublet due to insufficient ^{1}H decoupling.

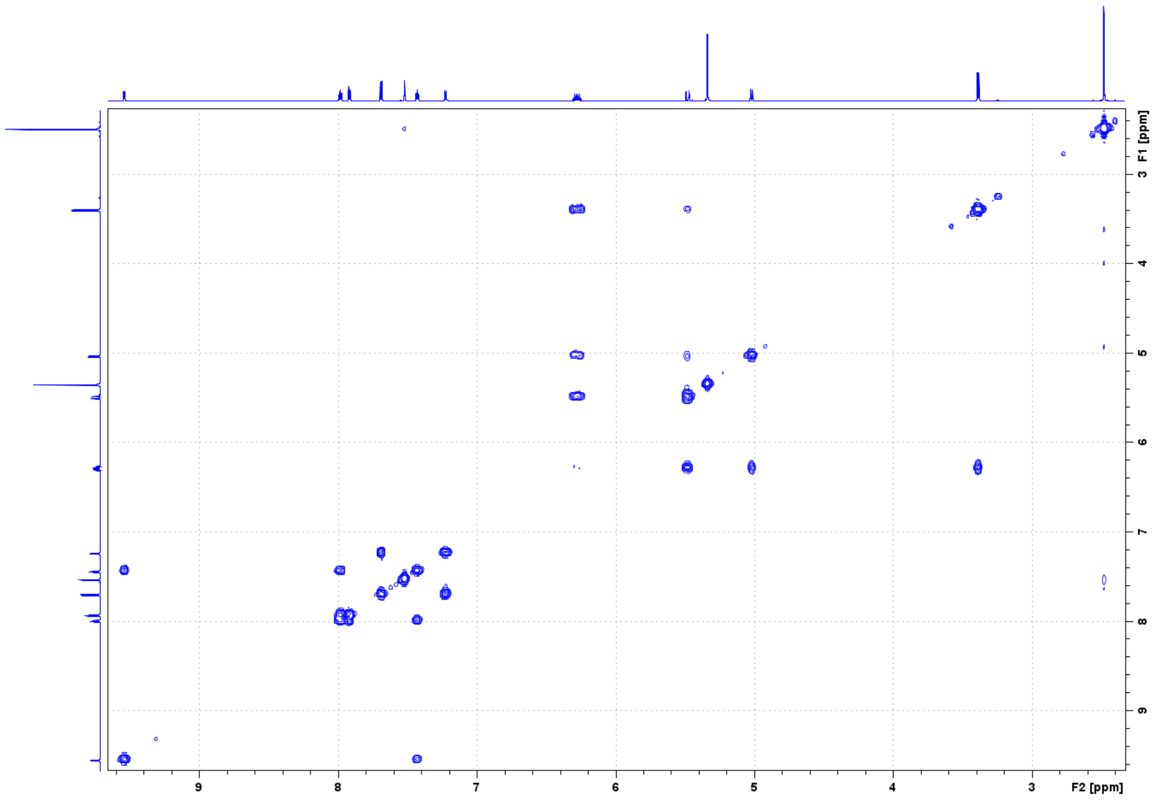

Figure S 3. COSY (800 MHz, CD_2Cl_2) spectrum of complex **2**.

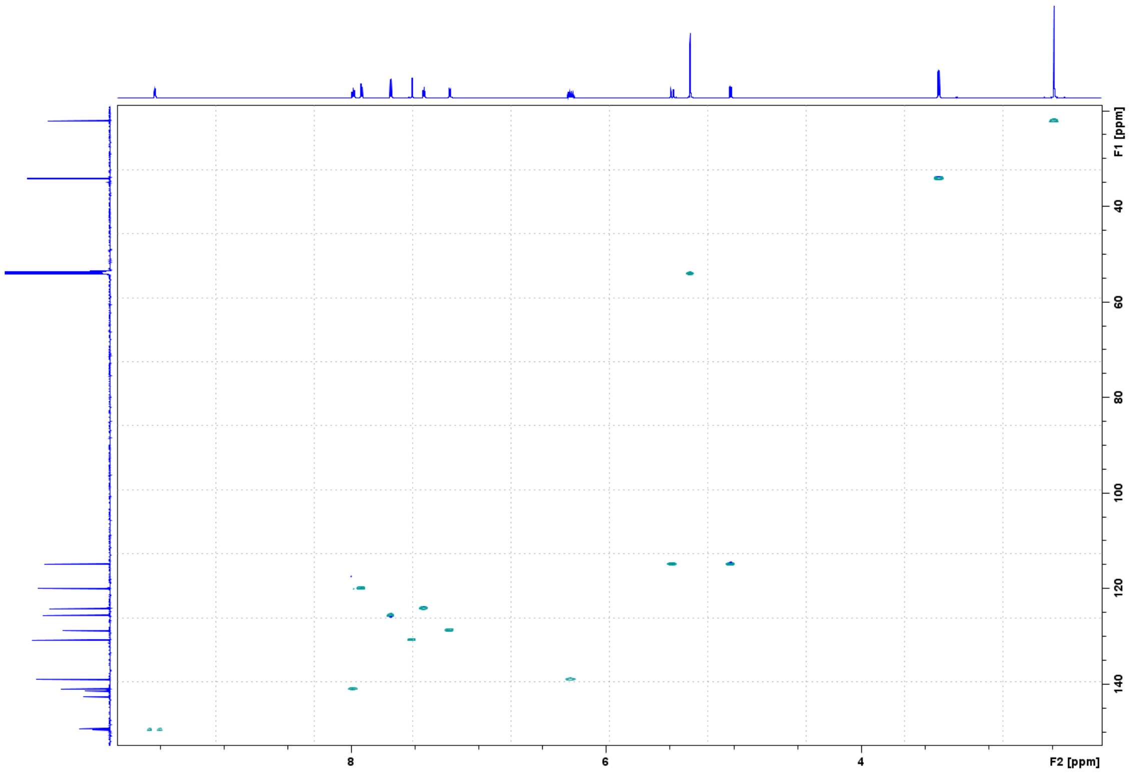

Figure S 4. HSQC (800 MHz, CD_2Cl_2) spectrum of complex **2**.

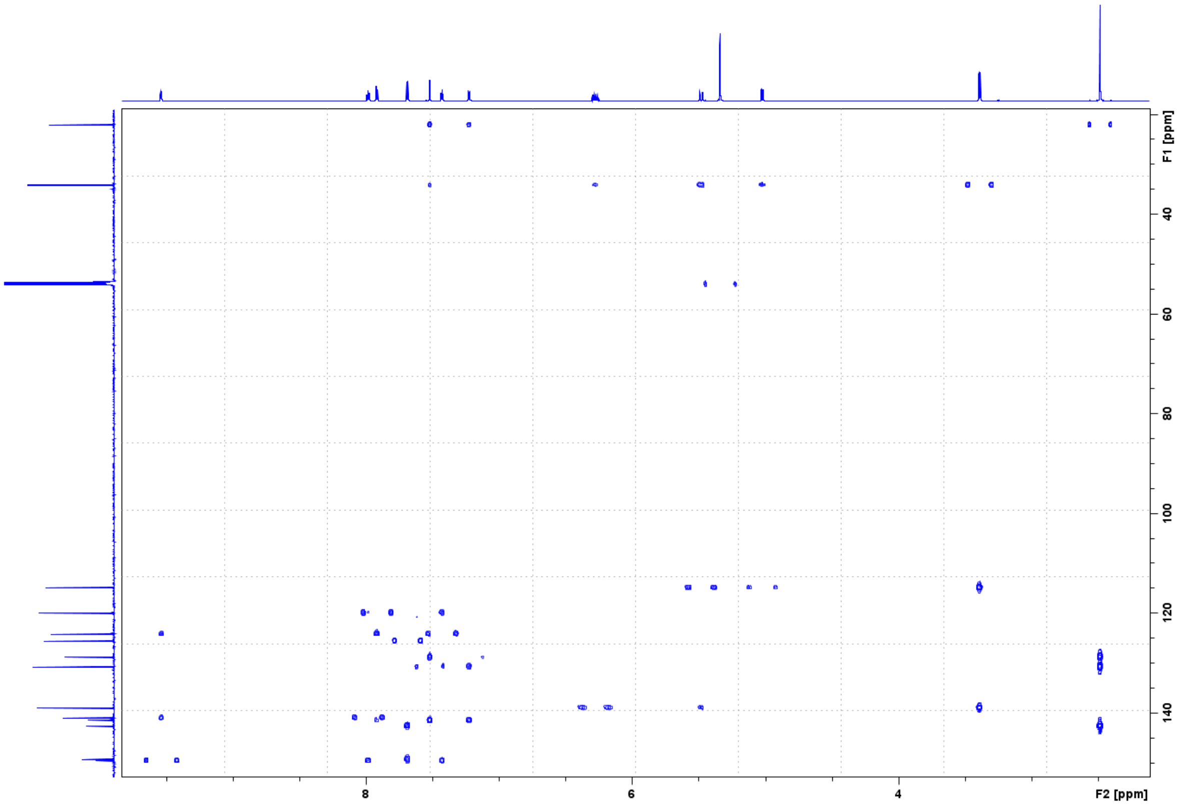

Figure S 5. HMBC (800 MHz, CD_2Cl_2) spectrum of complex **2**.

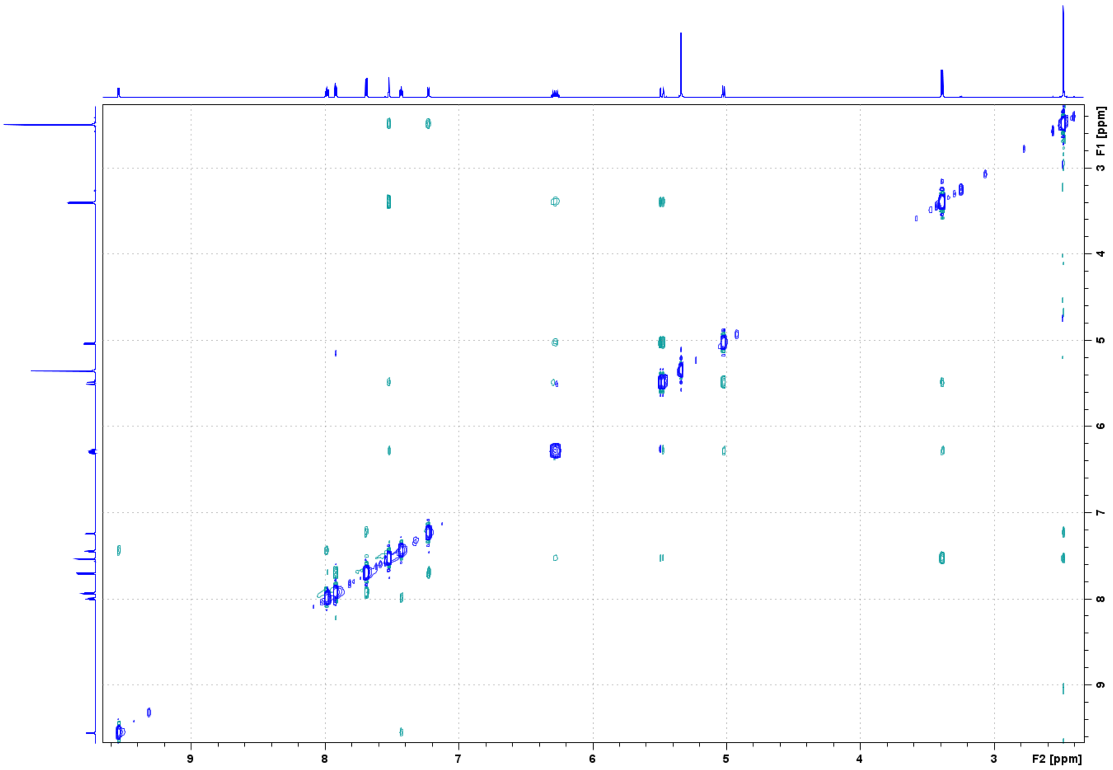

Figure S 6. NOESY (800 MHz, CD_2Cl_2, mixing time = 1 s) spectrum of complex **2**.

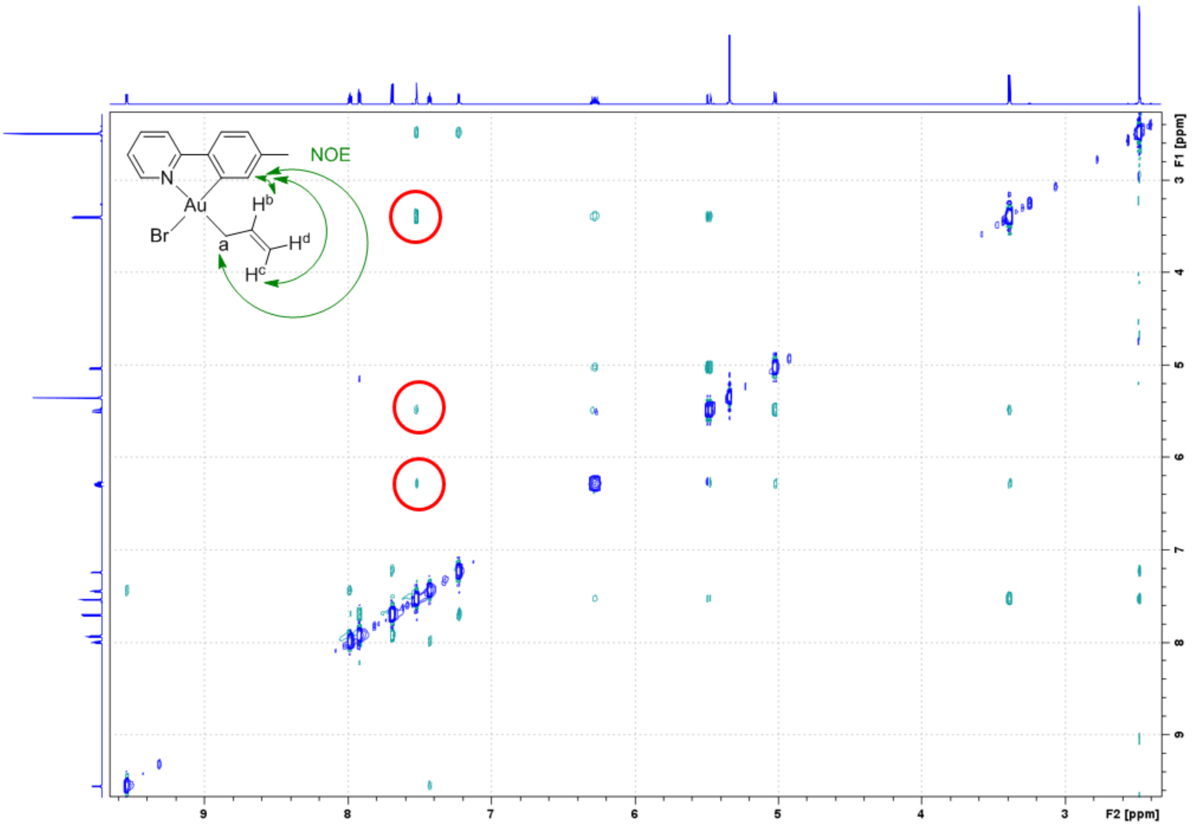

Figure S 7. NOESY (800 MHz, CD_2Cl_2, mixing time = 1 s) spectrum of complex **2**. Close up view on NOE between H^a, H^b, H^c and $H^{6'}$, showing that the allyl group is located *trans* to tpy-*N*.

NMR spectra of complex 3

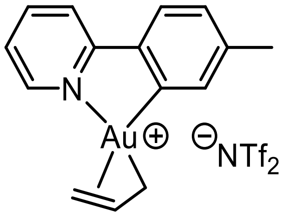

3

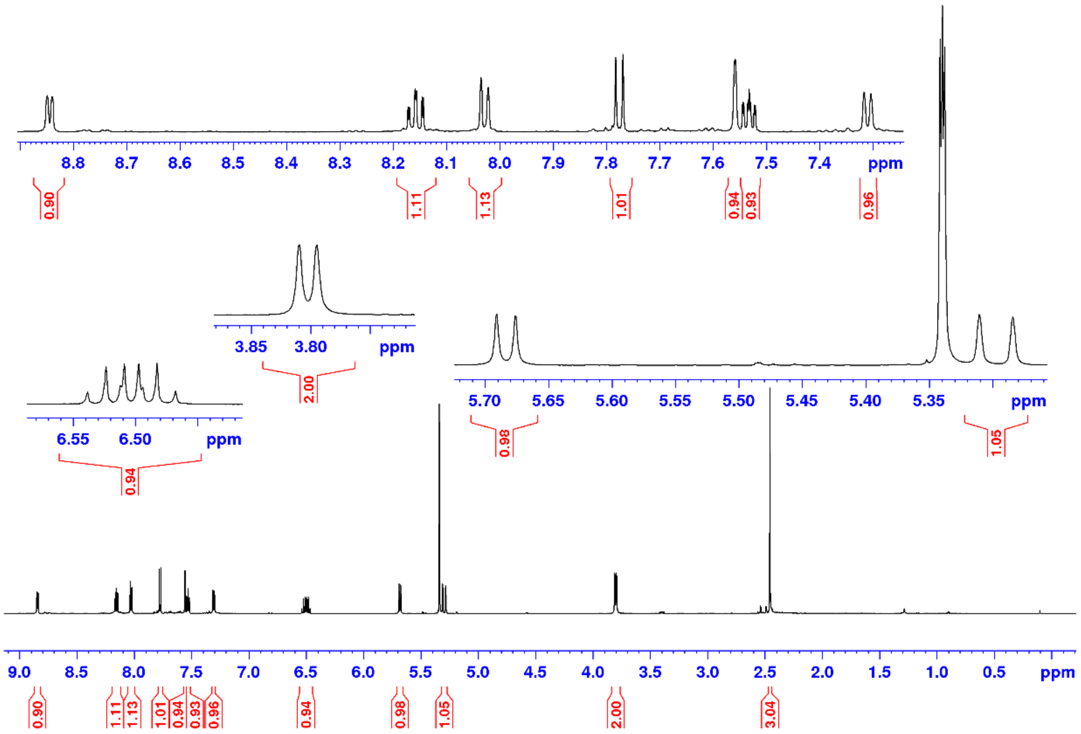

Figure S 8. ^{1}H NMR (600 MHz, CD_2Cl_2, 27 ^{o}C) spectrum of complex **3**.

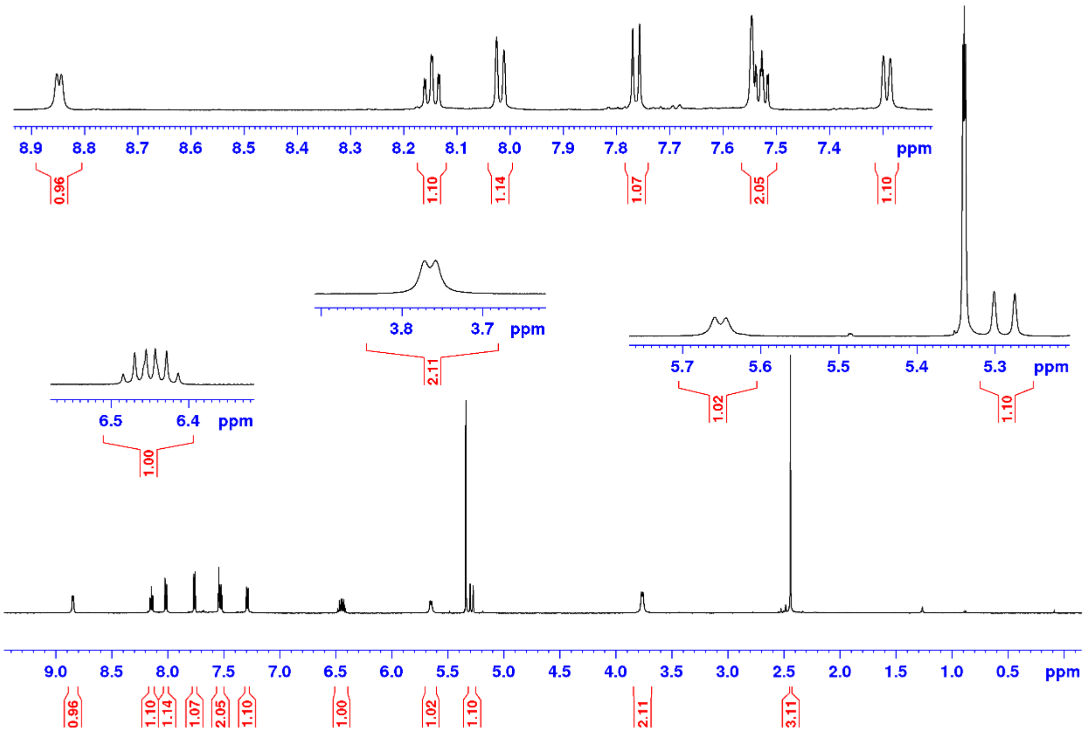

Figure S 9. ^1H NMR (600 MHz, CD_2Cl_2, 7 ^oC) spectrum of complex **3**.

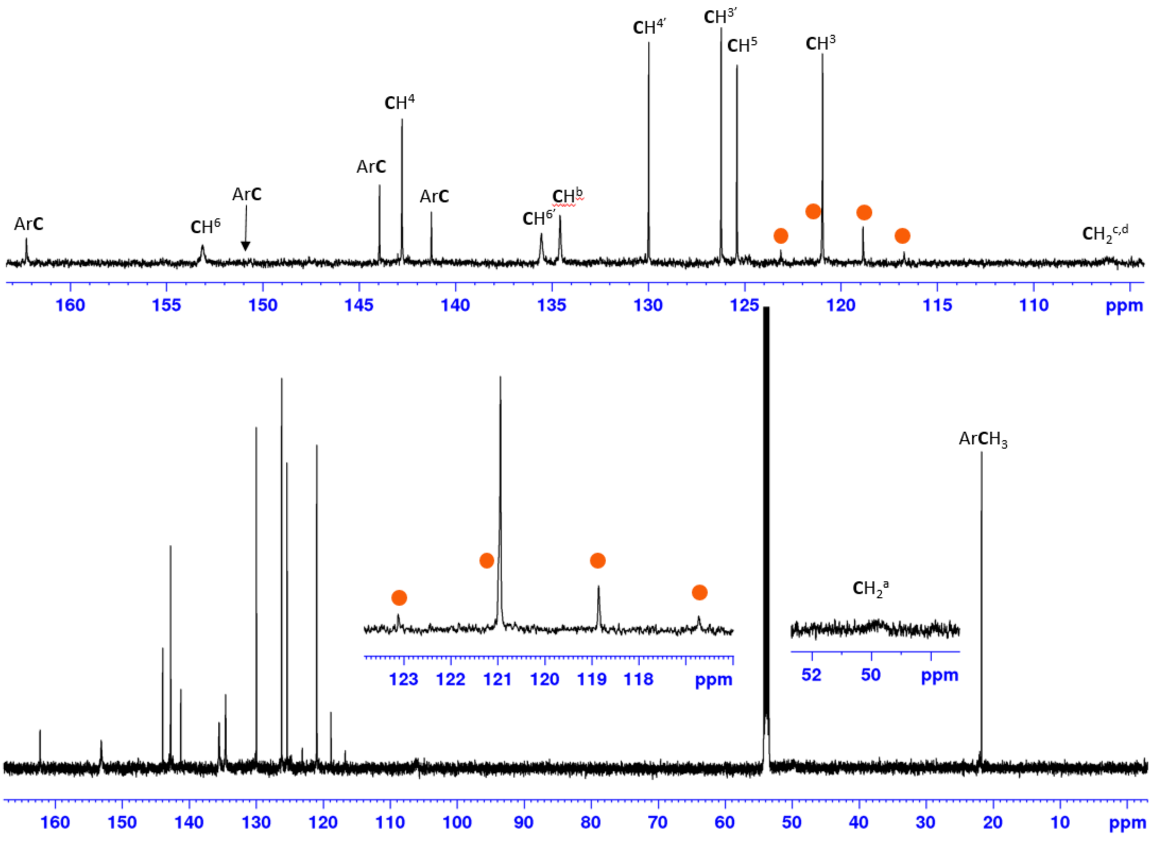

Figure S 10. ^{13}C NMR (151 MHz, CD_2Cl_2, ns = 3072, d1 = 4 s, 7 °C) spectrum of complex **3**. Several resonances are broadened (see insets). The quartet of CF_3, arising from coupling to ^{19}F, is marked with four orange circles.

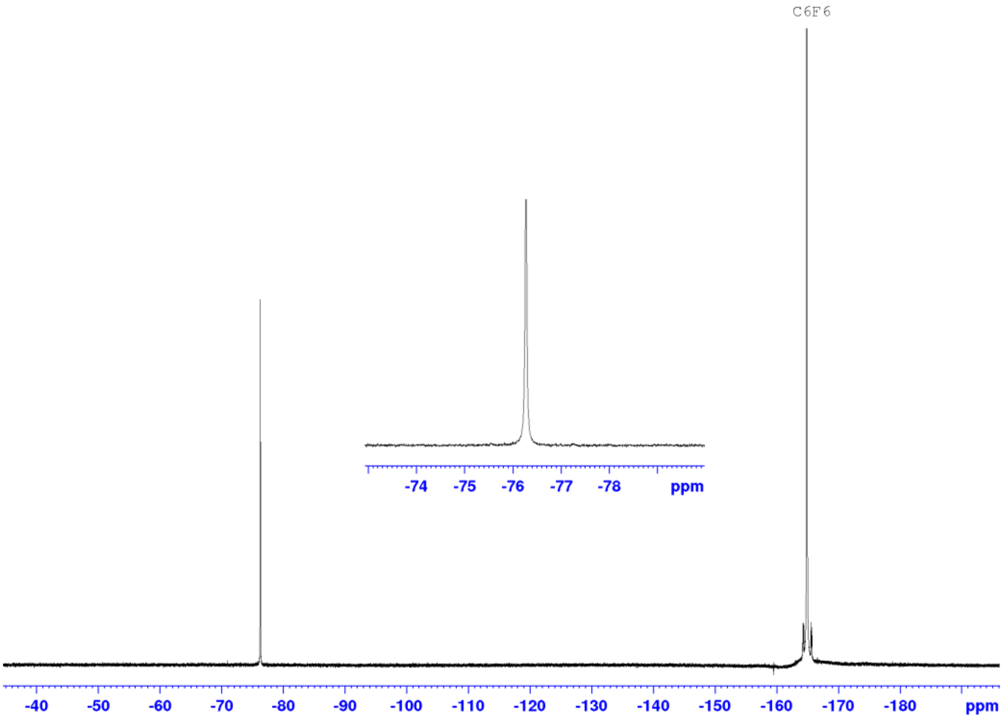

Figure S 11. ^{19}F NMR (188 MHz, CD_2Cl_2, 25 °C) of complex **3**.

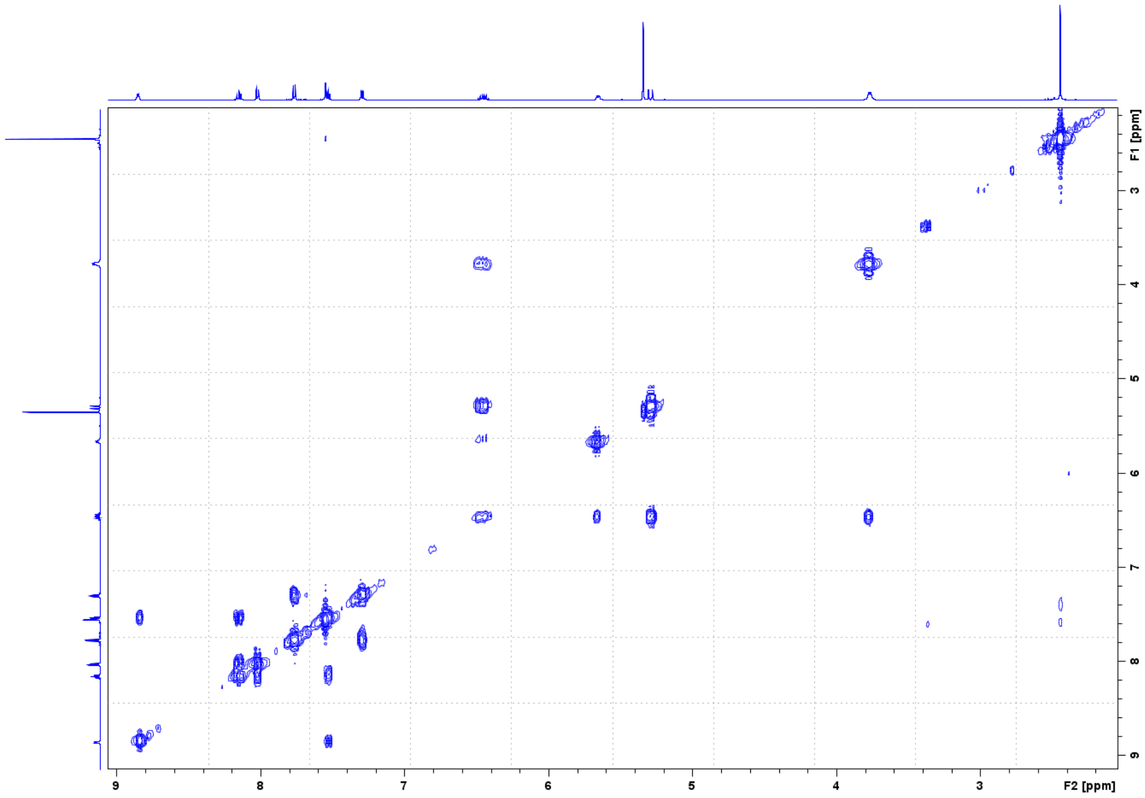

Figure S 12. COSY (600 MHz, CD_2Cl_2, 7 °C) spectrum of complex **3**.

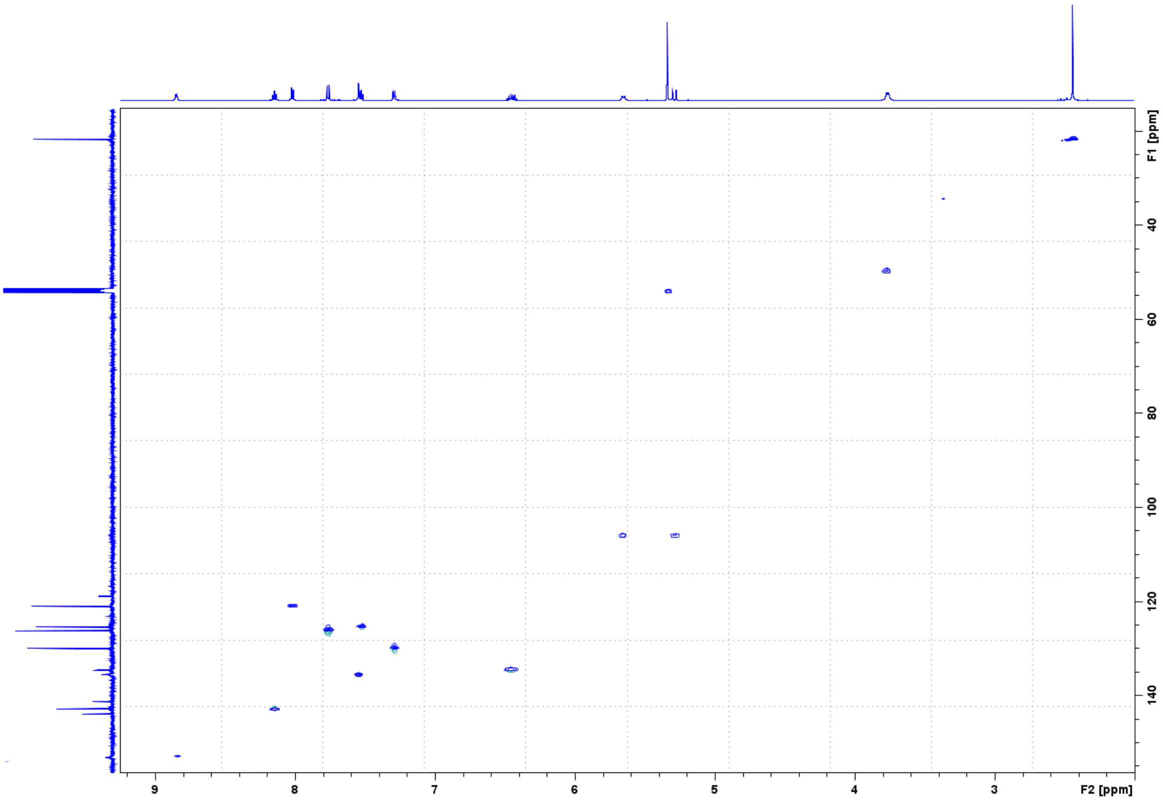

Figure S 13. HSQC (600 MHz, CD_2Cl_2, 7 °C) spectrum of complex **3**.

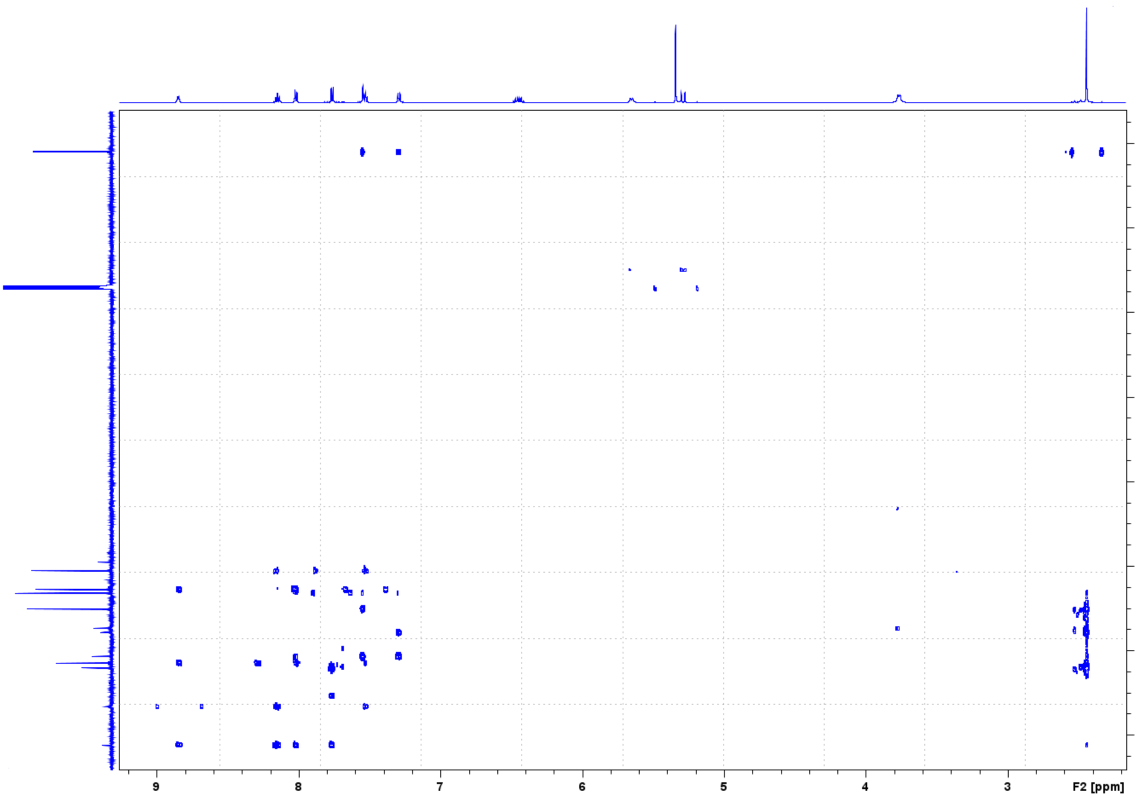

Figure S 14. HMBC (600 MHz, CD_2Cl_2, 7 °C) spectrum of complex **3**.

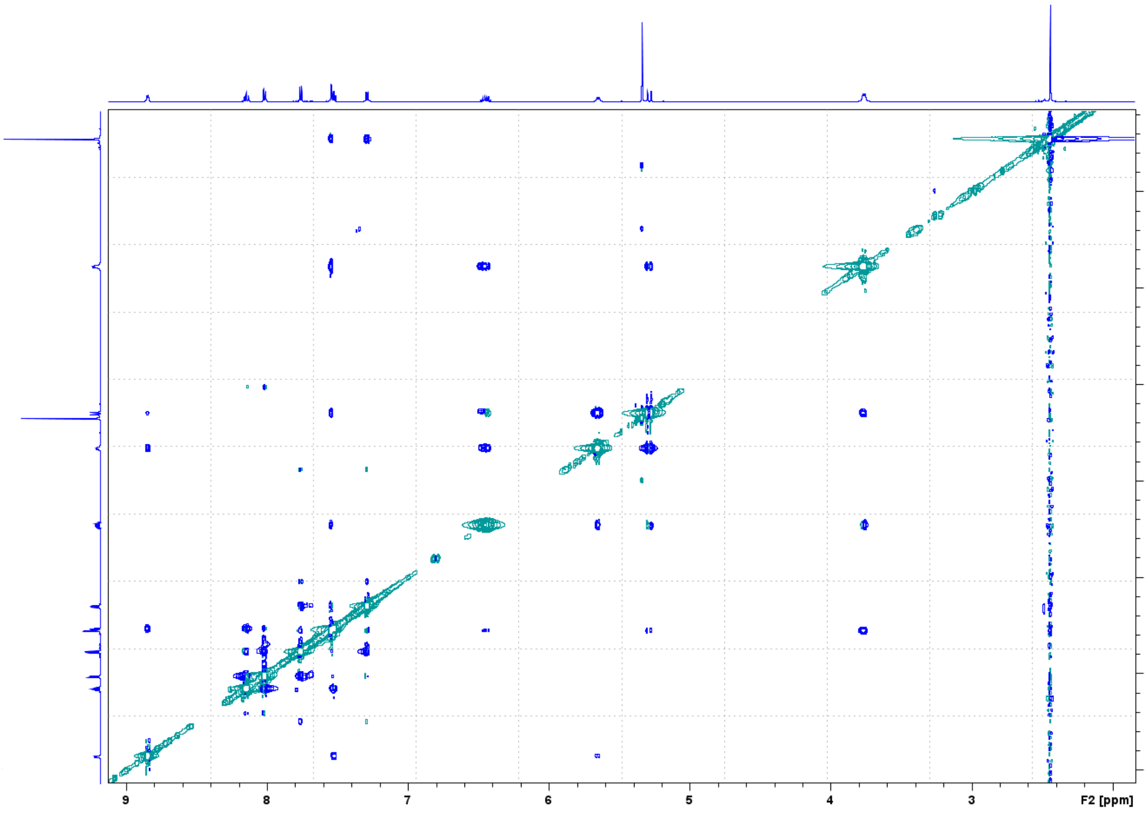

Figure S 15. NOESY (600 MHz, CD_2Cl_2, mixing time = 1 s, 7 °C) spectrum of complex **3**.

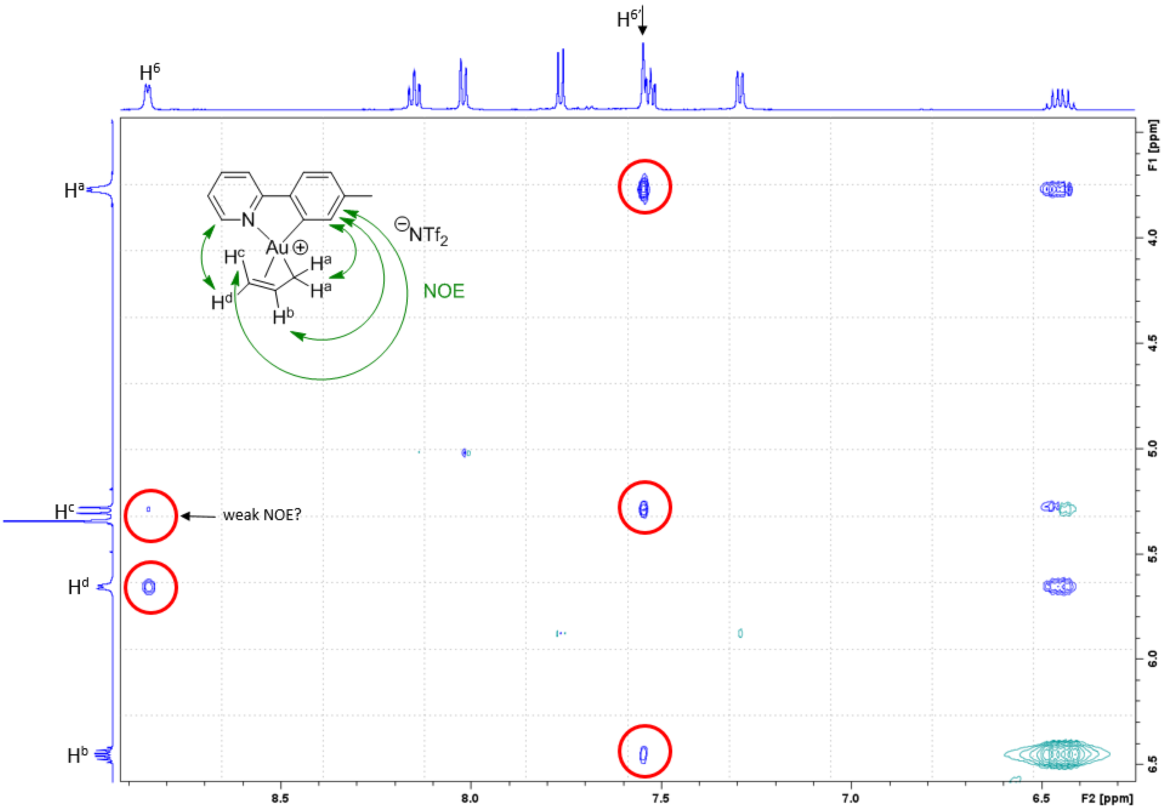

Figure S 16. ^1H-^1H NOESY (600 MHz, CD_2Cl_2, mixing time = 1 s, 7 °C) spectrum of complex **3**. Close up on the correlations between $H^{6'}$ and H^a, H^b and H^c and H^6 and H^d and H^c (weak).

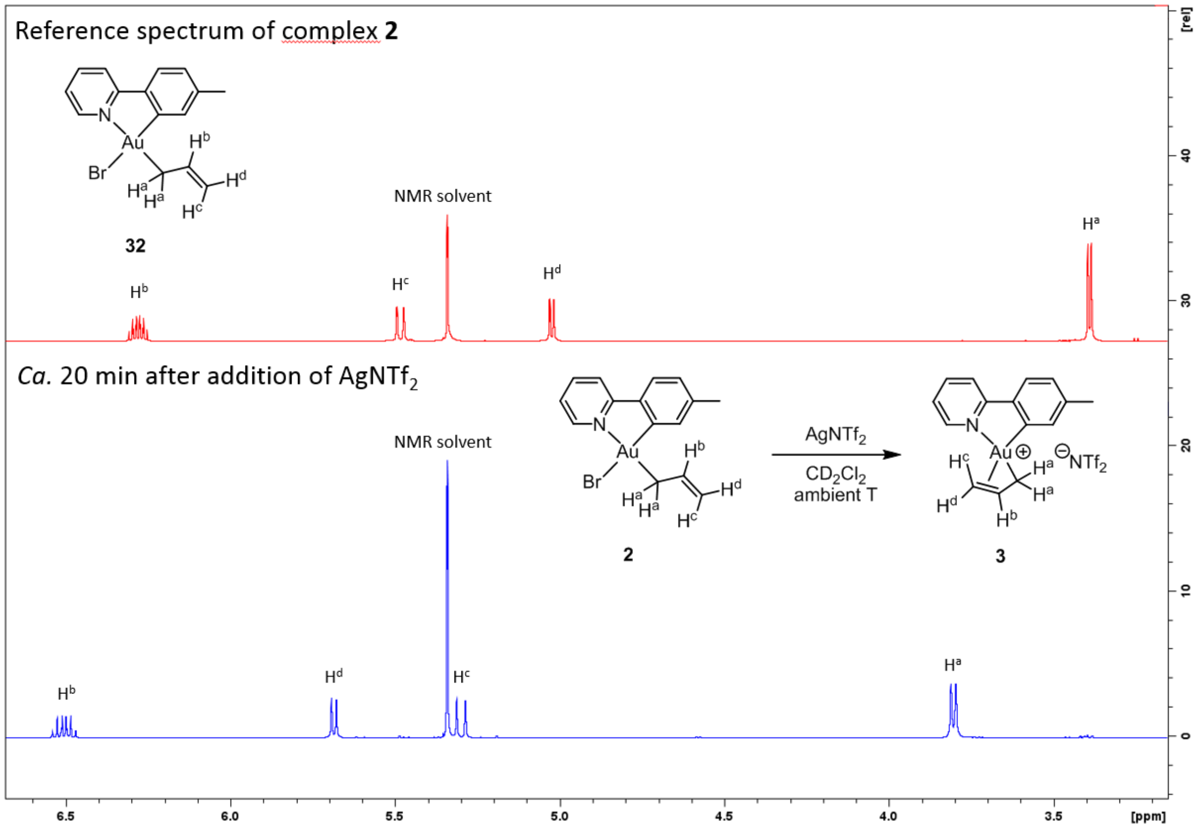

Figure S17. Stacked ^{1}H NMR (800 MHz (top) or 600 MHz (bottom), CD_2Cl_2) spectra of the reaction of **2** with $AgNTf_2$ forming complex **3**. Top: Reference spectrum of complex **2**. Bottom: Spectrum acquired *ca.* 20 min after addition of $AgNTf_2$. Only parts of the ^{1}H NMR spectra are shown.

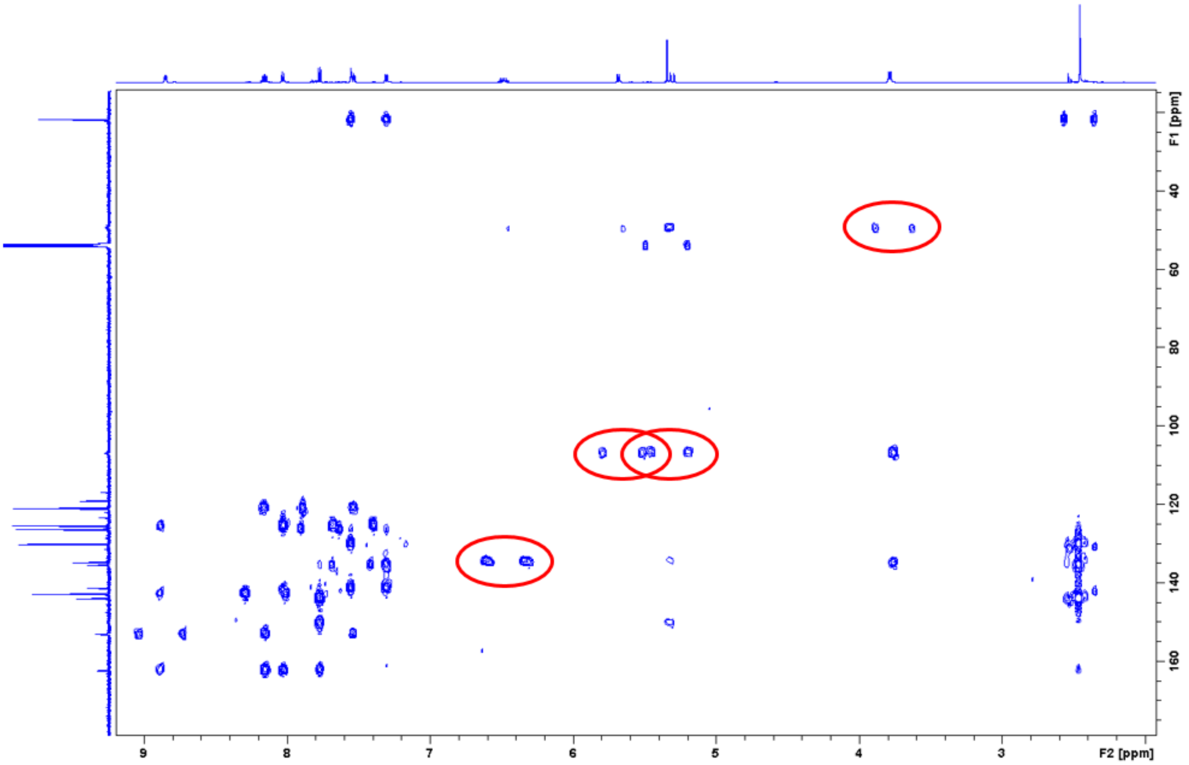

Figure S18. HMBC (600 MHz, CD_2Cl_2, 27 °C) of a partially decomposed sample of **3** used for determining ^{1}J (^{13}C–^{1}H).

NMR spectra of and [Au(η^1-allyl)(CD_3CN)(tpy)]$^+$[NTf_2]$^-$

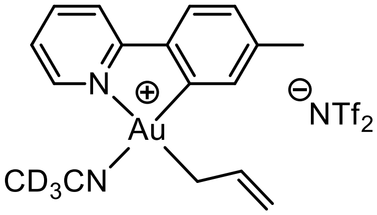

[Au(η^1-allyl)(CD_3CN)(tpy)]$^+$[NTf_2]$^-$

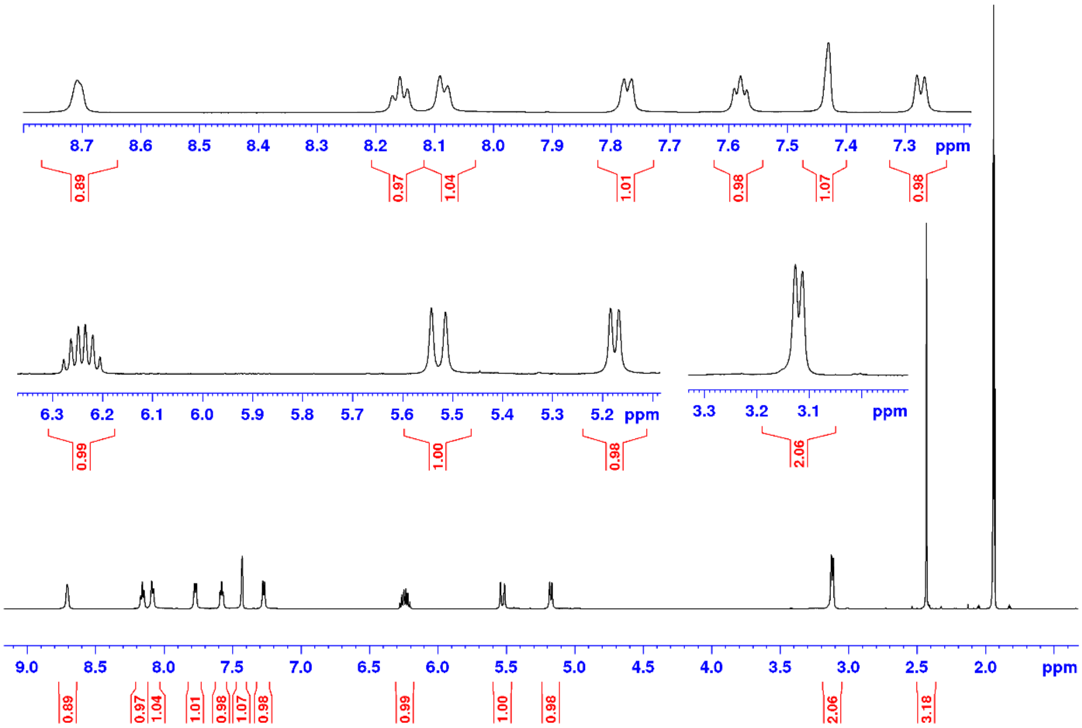

Figure S19. ^1H NMR (600 MHz, CD_3CN) spectrum of [Au(η^1-allyl)(CD_3CN)(tpy)]$^+$[NTf_2]$^-$.

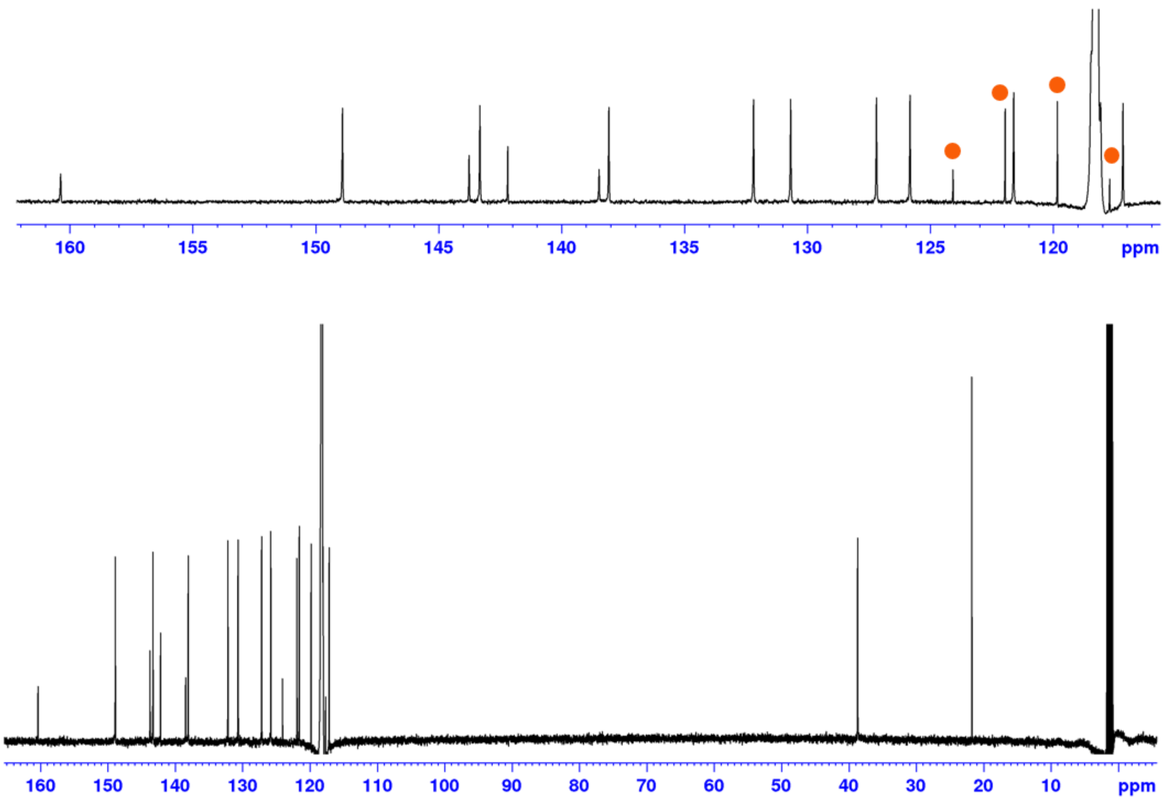

Figure S20. ^{13}CNMR (151 MHz, CD_3CN, ns = 7168, d1 = 5 s) spectrum of [Au(η^1-allyl)(CD_3CN)(tpy)]$^+$[NTf_2]$^-$. The quartet of CF_3, arising from coupling to ^{19}F, is marked with four orange circles.

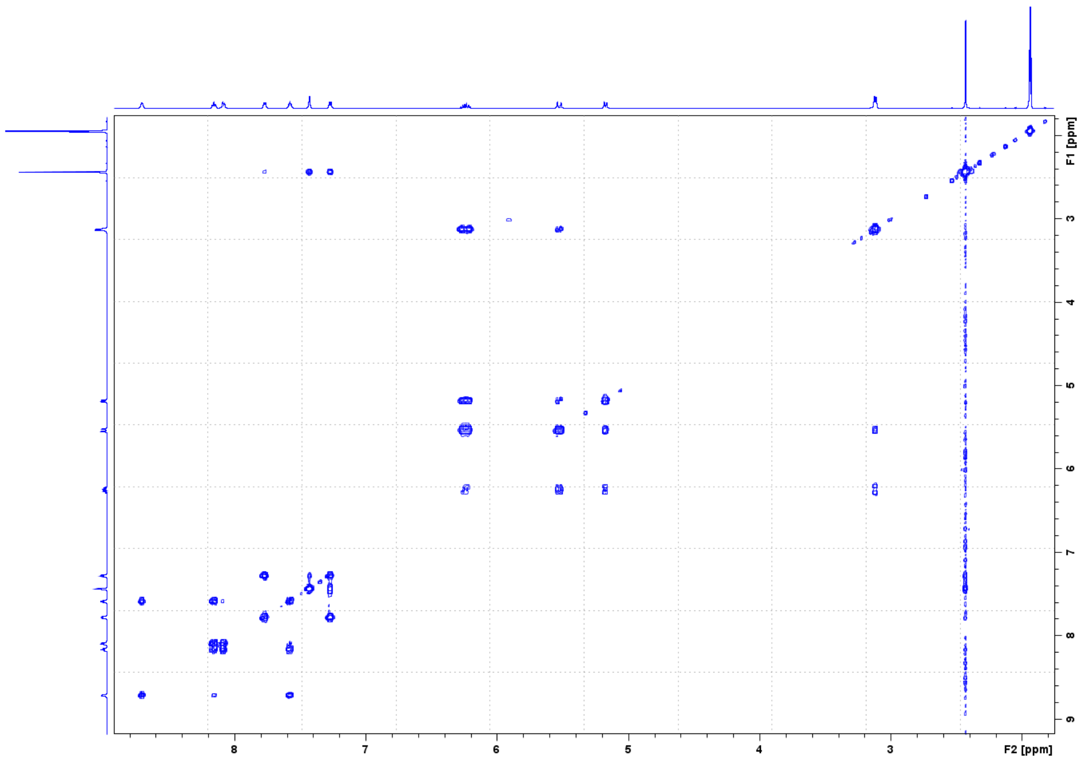

Figure S21. COSY (600 MHz, CD_3CN) spectrum of [Au(η^1-allyl)(CD_3CN)(tpy)]$^+$[NTf_2]$^-$.

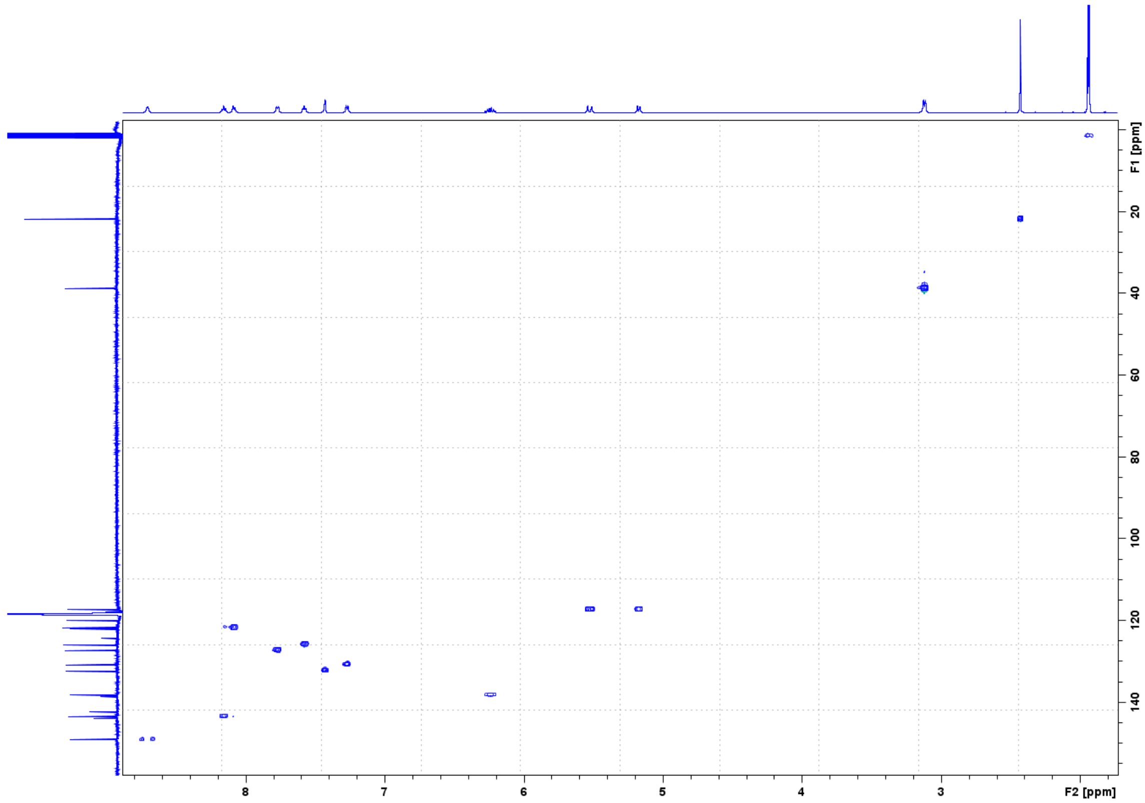

Figure S22. HSQC (600 MHz, CD_3CN) spectrum of [Au(η^1-allyl)(CD_3CN)(tpy)]$^+$[NTf_2]$^-$.

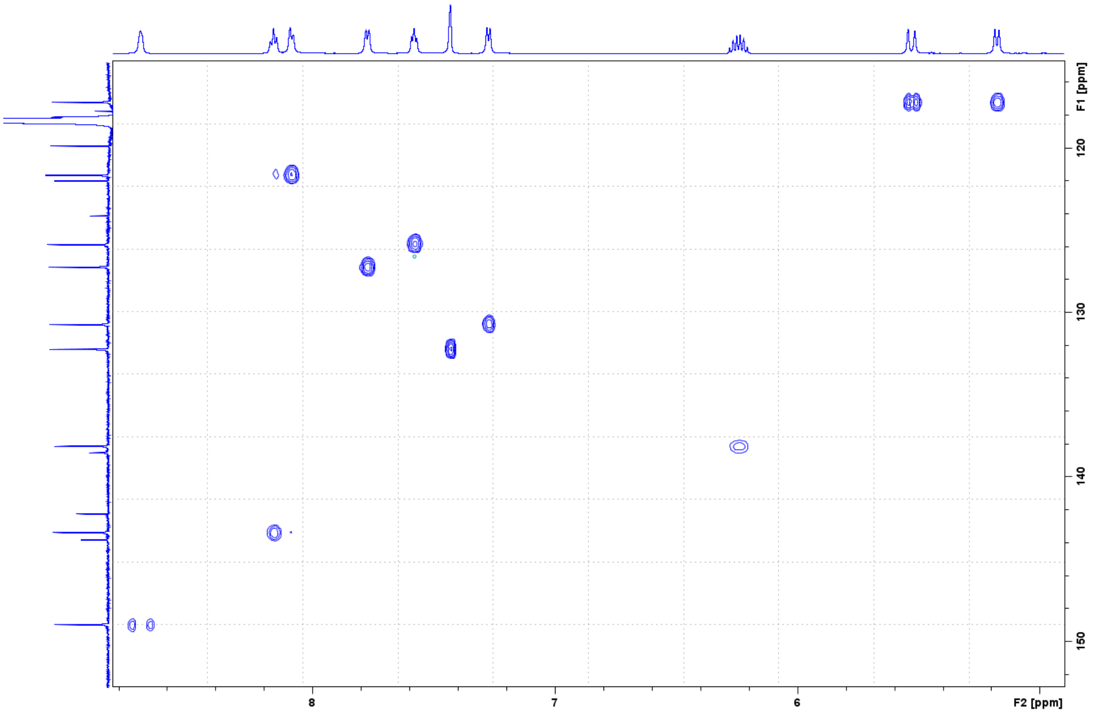

Figure S23. HSQC (600 MHz, CD_3CN) spectrum of [Au(η^1-allyl)(CD_3CN)(tpy)]$^+$[NTf_2]$^-$. Close up on the aromatic and vinylic region.

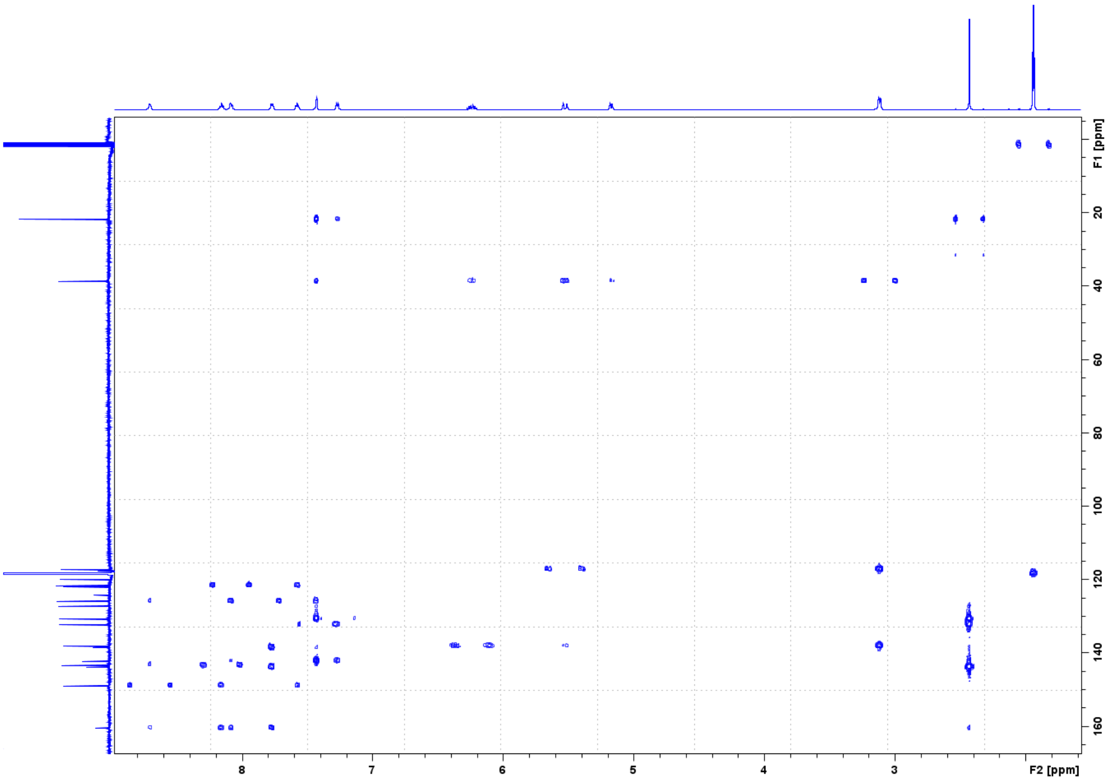

Figure S24. HMBC (600 MHz, CD_3CN) spectrum of [Au(η^1-allyl)(CD_3CN)(tpy)]$^+$[NTf_2]$^-$.

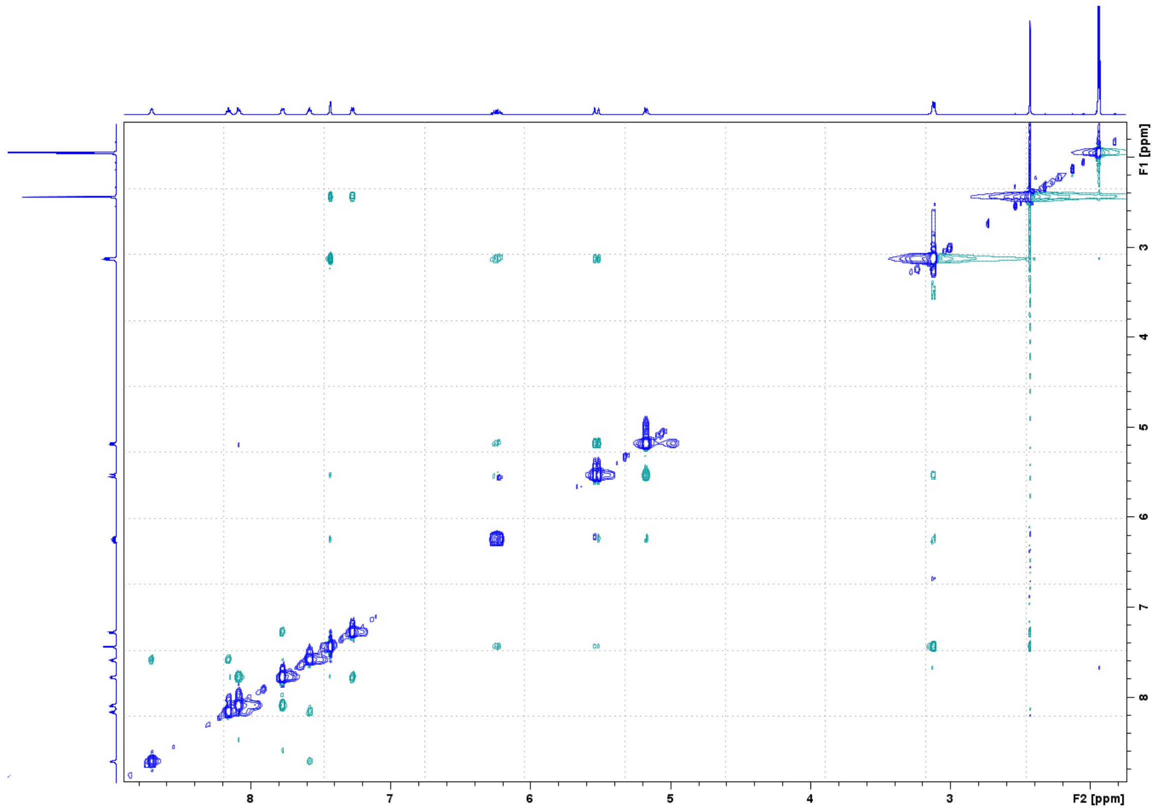

Figure S25. NOESY (600 MHz, CD_3CN, mixing time = 1 s) spectrum of $[Au(\eta^1\text{-allyl})(CD_3CN)(tpy)]^+[NTf_2]^-$.

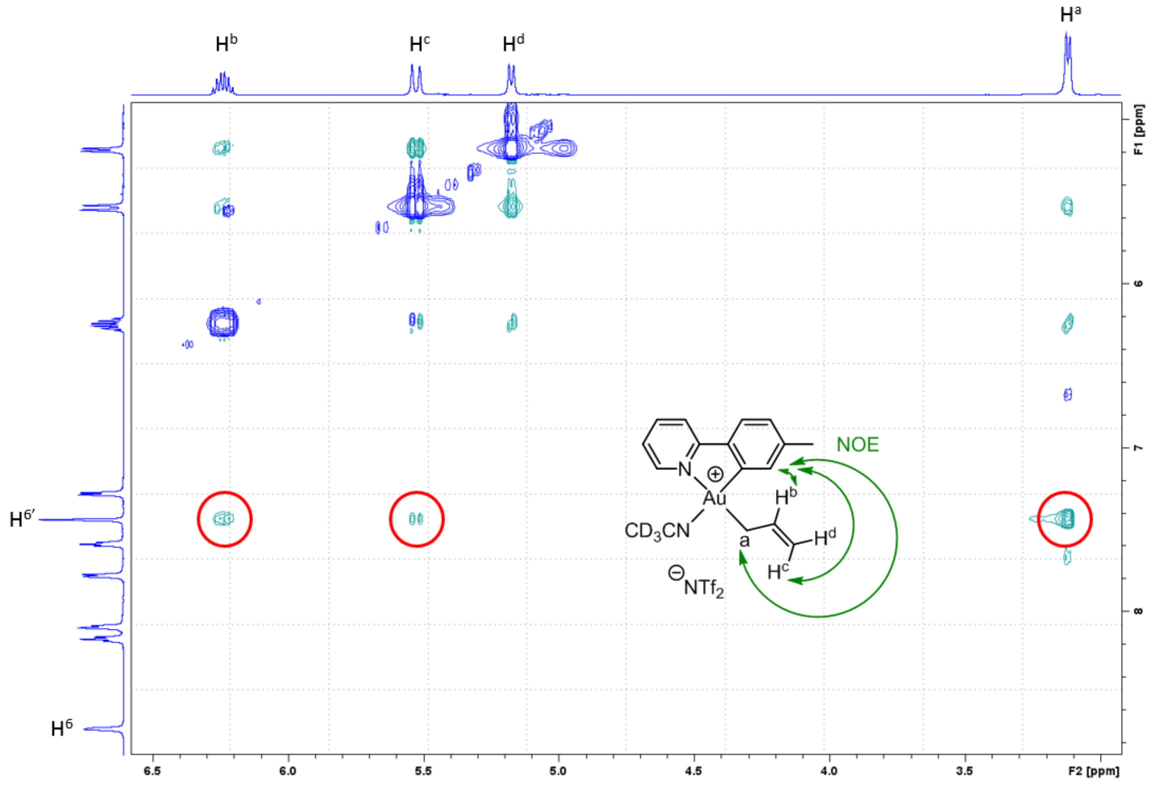

Figure S26. NOESY (600 MHz, CD_3CN, mixing time = 1 s) spectrum of $[Au(\eta^1\text{-allyl})(CD_3CN)(tpy)]^+[NTf_2]^-$. Close up view on the NOE between $H^{6'}$ and H^a, H^b and H^c, showing that the allyl group is located *trans* to tpy-*N*. No correlation between the allylic protons and H^6 were observed, indicating the formation of an η^1 allyl complex where CD_3CN probably is coordinated *trans* to tpy-*C*.

Variable temperature ^1H NMR of complex 3

Complex **3** was generated *in situ* as described previously (see page S4). The sample was kept at *ca.* 0 ^oC (ice bath) for a short time before the measurements. The sample was monitored by ^1H NMR (500 MHz, CD_2Cl_2) at various temperatures ranging from 17.2 ^oC to −79.2 (spectra at selected temperatures are shown in Figure S27) with intervals of *ca.* 10 ^oC. At each temperature, shimming of the magnetic field together with tuning and matching of the probe were performed. The temperature inside the probe was measured after each measurement using a Delta OHM HD9214 thermometer fitted inside a NMR tube containing CD_2Cl_2. Some decomposition of the sample occurred during the measurements (see Figure S27).

Variable temperature ^{1}H NMR spectra of complex 3

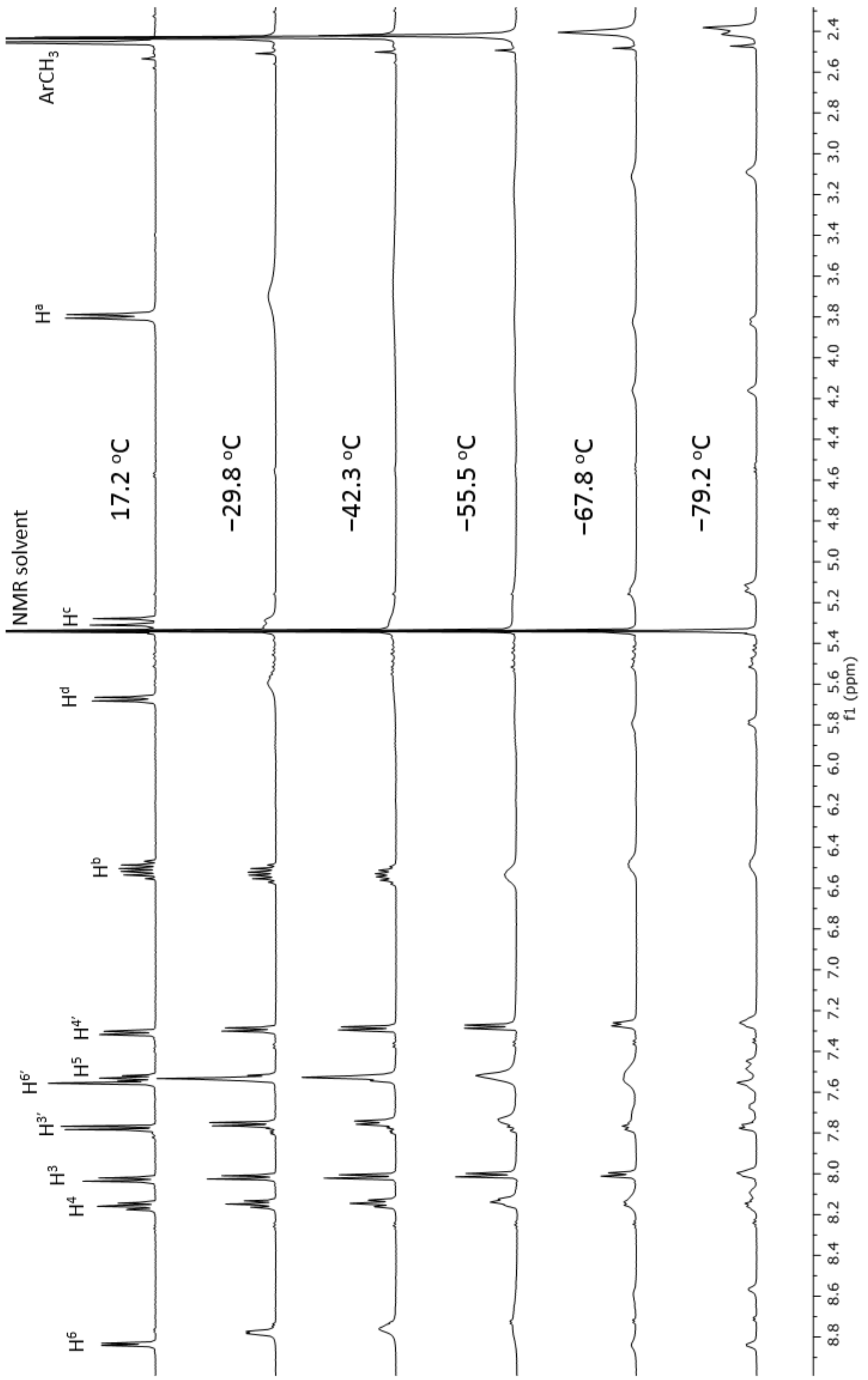

Figure S27. Low temperature ^{1}H NMR (500 MHz, CD_2Cl_2) spectra of the allylic region of **3**. Some minor peaks are due to slow decomposition.

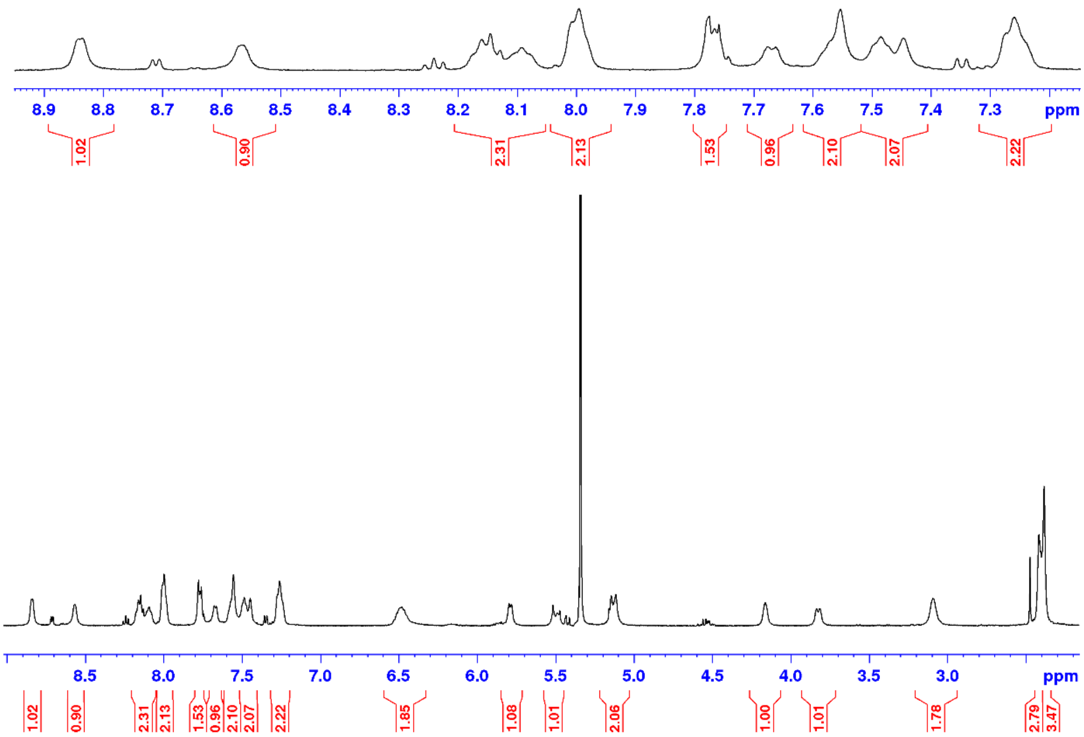

Figure S 28. ^{1}H NMR (500 MHz, CD_2Cl_2) spectrum of **3** at −79.2 ºC. Some minor peaks are due to decomposition.

Crystallographic structure determination

Data sets for complexes **2** and **3** were obtained at the Swiss-Norwegian beamline (SNBL, BM01)[2] at the European Synchrotron Radiation Facility (ESRF) in Grenoble, France. Data collection, cell refinement and data reduction was performed using CrysAlis PRO.[3] The structures were solved with ShelxT[4] and refined with ShelxL.[5] Olex2[6] was used as user interface. The cif files were edited with enCIFer v1.4,8[7] and molecular graphics were produced with Diamond v4.1.2.9[8] For complex **3**, two crystals were measured and the two data sets were merged after data reduction using least-squares fitting of common reflections in XPREP.[9] The measurements at ESRF were performed with help from Dr. Dmitry Chernyshov (SNBL, ESRF). Twinning occurs in complex **3**. The twin law was resolved using TwinRotMat,[10] and the structures were refined using de-twinned data sets. All metrical data are contained in the respective cif files, available from https://www.ccdc.cam.ac.uk/ (CCDC numbers 1936342-1936343).

Complex 3

Complex **3** was generated *in situ* from complex **2** (5 mg, 0.01 mmol, 1 equiv) and $AgNTf_2$ (7 mg, 0.02 mmol, 2 equiv) in CH_2Cl_2 (1-2 mL) inside an argon filled glove box. After 1-2 minutes, the solution was filtrated and the filtrate was distributed between two small vials. Each of the vials were placed in one capped larger vial each containing pentane, and placed in a freezer inside the glove box (–36 ^oC) slowly yielding crystals of **3**. The vials were taken out of the freezer and the solvent was carefully removed with a pipette. The crystals were submerged in Parabar 10312, a viscous cryoprotectant oil to protect them from moisture and air, and the vials were again closed. The vials were taken out of the glovebox and placed in a dry ice-cooled dewar. The crystals were mounted in a cold room at 4 ^oC with help from Dr. Kaare Bjerregaard-Andersen (Bio^3- Chemical Life Sciences, University of Oslo) and stored in a dewar cooled by liquid nitrogen which was shipped to ESRF. The crystals were kept in the cooled dewar until they were measured.

Complex 2

Crystals suitable for single crystal X-ray diffraction analysis of complex **2** were obtained by placing a solution of **2** in a small vial placed inside a capped larger vial containing pentane. The vial was placed inside a freezer (–36 ^oC) slowly yielding crystals of **2**. The crystals of **2** were also measured at ESRF and for simplicity, they were mounted and shipped the same way as the crystals of **3**, even though complex **2** is not sensitive to air or moisture.

Crystallographic structure determination of complex 2

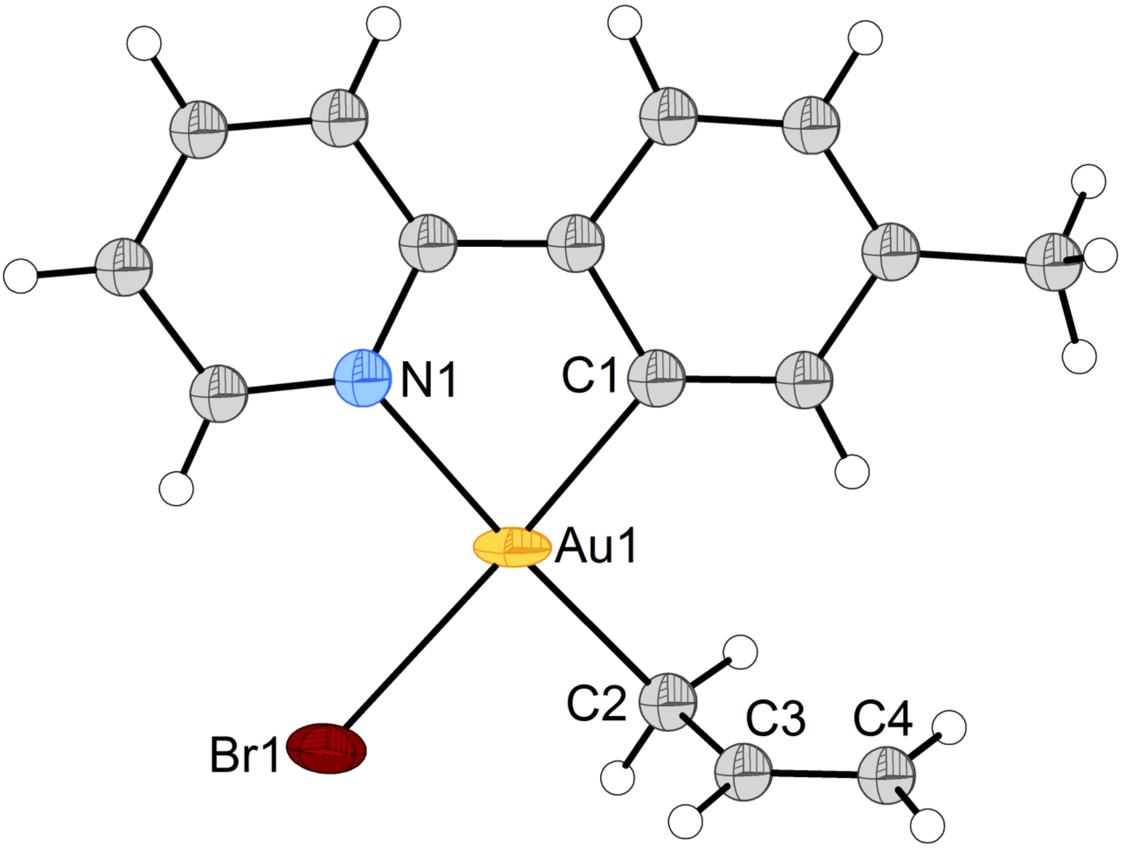

Figure S29. ORTEP plot of complex **2**. Due to twinning and disorder limiting the high resolution diffraction in the measured crystal, only Au and Br are refined as ellipsoids (at 50%). Selected bond lengths [Å] and bond angles [º]: Au1-N1, 2.11(3); Au1-C1, 2.02(4); Au1-C2, 2.10(4); Au1-Br1, 2.493(5); C2-C3, 1.41(5); C3-C4, 1.31(5); Br1-Au1-N1, 95.0(9); N1-Au1-C1, 81.7(15); C1-Au1-C2, 94.9(16); C2-Au1-Br1, 88.7(12); C1-Au1-Br1, 174.9(12); N1-Au1-C2, 174.1(15); Au1-C2-C3, 114(3); C2-C3-C4, 126(4).

Crystallographic structure determination of complex 3

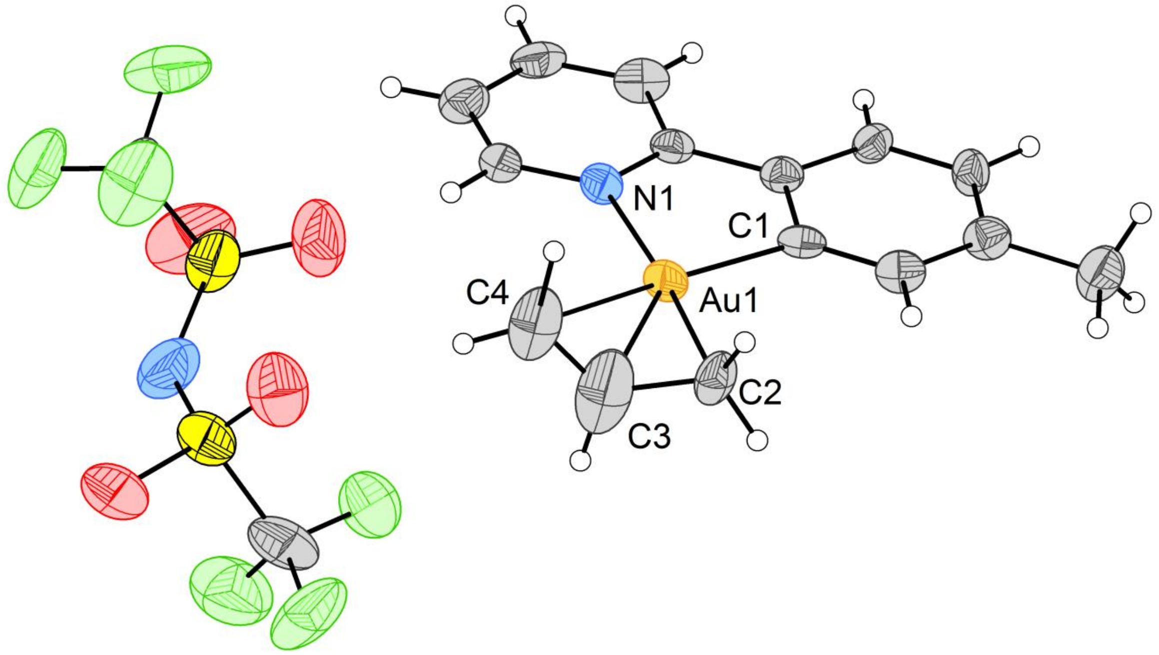

Figure S30. ORTEP plot of complex **3** with 50% ellipsoids. Selected bond lengths [Å] and bond angles [°]: Au1-N1, 2.119(16); Au1-C1, 2.04(2); Au1-C2, 2.062(19); Au1-C3, 2.21(2); Au1-C4, 2.35(2); C2-C3, 1.43(3); C3-C4, 1.22(4); C4-Au1-N1, 109.8(8); N1-Au1-C1, 81.0(7); C1-Au1-C2, 104.1(8); C2-Au1-C4, 65.2(8); C1-Au1-C4, 166.3(9); N1-Au1-C2, 174.9(8); Au1-C2-C3, 76.1(13); C2-C3-C4, 129(3).

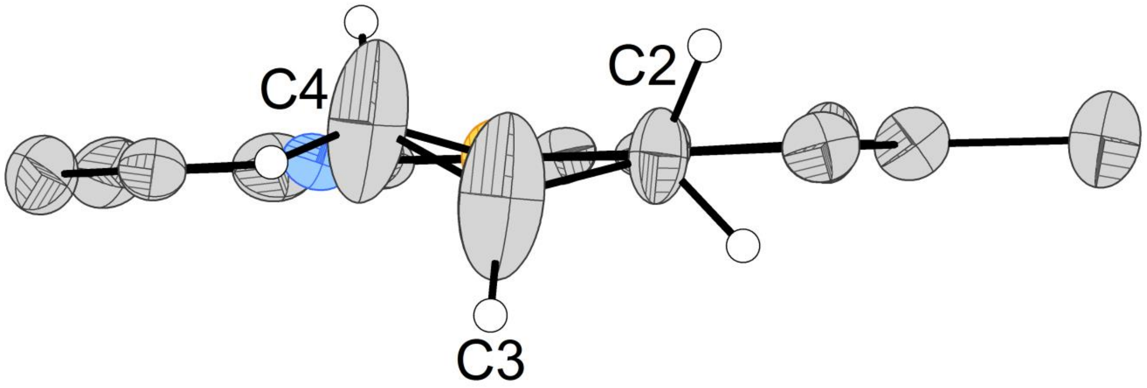

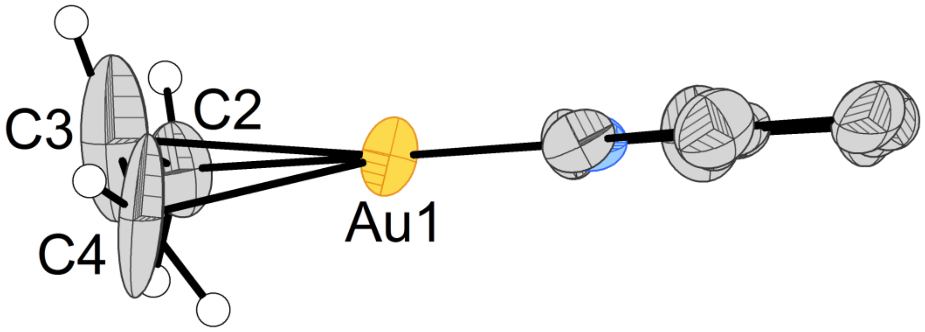

Figure S31. ORTEP plots of complex **3** with 50% ellipsoids. The NTf$^-$ anion has been removed for clarity.

Table 1. Crystal and refinement data for Au(III) complex **2**.

	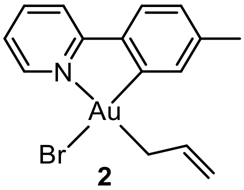 **2**
**Crystal data**	
Chemical formula	$C_{15}H_{15}AuBrN$
M_r	486.16
Crystal system, space group	Orthorhombic, *Pbcn*
Temperature (K)	100
a, *b*, *c* (Å)	9.5122(19), 7.7640(16), 37.770(8)
α, β, γ (°)	90, 90, 90
V (Å^3)	2789.4(10)
Z	8
Radiation type	Synchrotron radiation, λ = 0.78487 Å
μ (mm^{-1})	17.24
Crystal size (mm)	0.06 x 0.03 x 0.01
**Data Collection**	
Diffractometer	BM01-ESRF[2]
Absorption correction	Muliti-scan. CrysAlisPro 1.171.38.41 (Rigaku Oxford Diffraction, 2015). Empirical absorption correction using spherical harmonics, implemented in SCALE3 ABSPACK scaling algorithm.
T_{min}, T_{max}	0.502, 1.000
No. of measured, independent and observed [I > 2σ(I)] reflections	16792, 1090, 911
R_{int}	0.121
(sin θ/λ)max (Å^{-1})	0.454
**Refinement**	
$R[F^2 > 2\sigma(F^2)]$, $wR(F^2)$, S	0.100, 0.250, 1.22
No. of reflections	1090
No. of parameters	69
No. of restraints	0
H-atom treatment	H-atom parameters constrained
	$w = 1/[\sigma^2(F_o^2) + (0.1113P)^2 + 296.2275P]$ where $P = (F_o^2 + 2F_c^2)/3$
$\Delta\rho_{max}$, $\Delta\rho_{min}$ (e Å^{-3})	4.17, −1.61

Table 2. Crystal and refinement data for Au(III) complex **3**.

	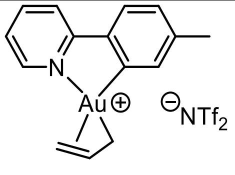<br>**3**
**Crystal data**	
Chemical formula	$C_{15}H_{15}AuN{\cdot}C_2F_6NO_4S_2$
M_r	686.40
Crystal system, space group	Monoclinic, $P2_1/n$
Temperature (K)	100
a, *b*, *c* (Å)	6.8423(4), 12.3649(7), 24.7602(11)
α, β, γ (°)	90, 96.956(4), 90
V ($Å^3$)	2079.40 (19)
Z	4
Radiation type	Synchrotron radiation, λ = 0.78487 Å
μ (mm^{-1})	9.47
Crystal size (mm)	0.04 x 0.03 x 0.01
**Data Collection**	
Diffractometer	BM01-ESRF[2]
Absorption correction	Multi-scan. CrysAlis PRO 1.171.38.46 (Rigaku Oxford Diffraction, 2015) Empirical absorption correction using spherical harmonics, implemented in SCALE3 ABSPACK scaling algorithm.
No. of measured, independent and observed [I > 2σ(I)] reflections	2169, 2169, 1842
R_{int}	0.100
(sin θ/λ)max ($Å^{-1}$)	0.505
**Refinement**	
$R[F^2 > 2\sigma(F^2)]$, $wR(F^2)$, S	0.069, 0.146, 1.25
No. of reflections	2169
No. of parameters	291
No. of restraints	6
H-atom treatment	H-atom parameters constrained
	$w = 1/[\sigma^2(F_o^2) + (0.0237P)^2 + 43.8297P]$ where $P = (F_o^2 + 2F_c^2)/3$
$\Delta\rho_{max}$, $\Delta\rho_{min}$ (e $Å^{-3}$)	1.96, −1.26

Reaction pathways for the interconversion of the η^3-allyl enantiomers 3 and 3'

Three different processes were found to yield **3'** from **3** (Figure S32): (a) counter clockwise rotation of the Au-C bond in **3** (**TS3-4**) followed by C-C rotation (**TS4-5**) and allyl recoordination via clockwise rotation of the Au-C bond in **5** (**TS5-3'**); (b) the same process but with opposite chirality, starting from the clockwise rotation of the Au-C bond in 3; (c) via a Cs symmetric TS which interconverts **3** to **3'** in one step.

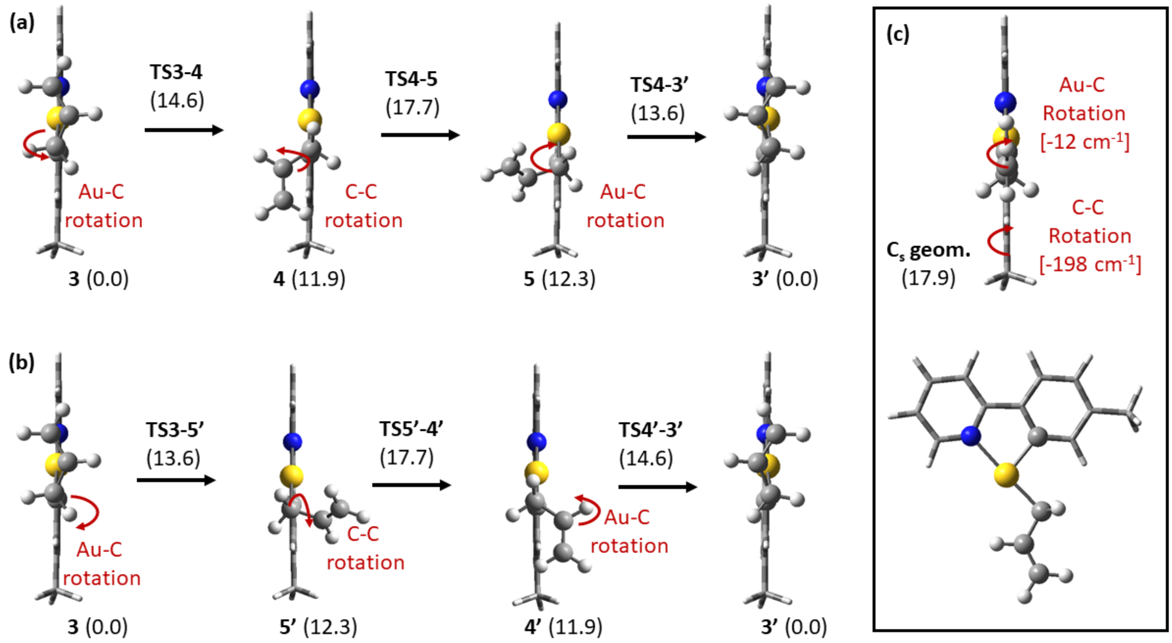

Figure S32. Asymmetric (a) and (b), and symmetric (c) reaction pathways for the interconversion of **3** and **3'**. Gibbs energies in solution (SMD) are given in kcal mol^{-1}.

Reaction pathway for the interconversion of the η^1-allyl intermediates 4 and 5 *via* coordination of the NTf_2 anion.

Coordination of the NTf_2 anion in the vacant site of intermediates **4** and **5** is exergonic by more than 10 kcal mol^{-1} yielding **4-NTf$_2$** and **5-NTf$_2$** respectively (Figure S33). The C2-C3 bond rotation from these intermediates is almost barrierless ($\Delta G^{\ddagger}$ = 2.8 kcal mol^{-1}).

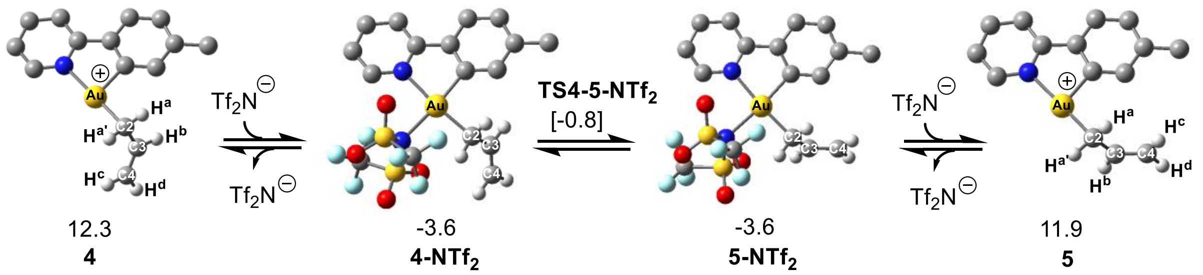

Figure S33. Reaction pathway for the η^1-allyl C-C bond rotation via coordination of the NTf_2 anion. Gibbs energies in solution (SMD) are given in kcal mol^{-1}.

M(η^3-allyl)(tpy) with M = Pt(II) and Au(III)

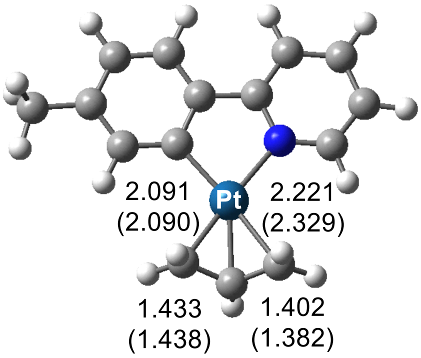

Figure S 34. Optimized geometry for Pt(η^3-allyl)(tpy). Pt-C and C-C bond distances in Å. In parentheses, distances obtained for Au(η^3-allyl)(tpy).

Computational details

Calculations were carried out at the DFT level as implemented in the Gaussian09 software package.[11] The hybrid PBE0+GD3 functional[12,13] including Grimme's model for dispersion forces was used to optimize all geometries. This methodology was selected because previous studies have proven its solid performance in the modeling of Au(III) complexes.[14] C, H, N, O, S and F were described with the all-electron triple-ζ 6-311+G** basis set,[15] whereas Au was described with the Stuttgart–Köln basis set including a small-core quasi-relativistic pseudopotential.[16] Geometries were fully optimized without any constraint. Vibrational frequencies were computed at the same level of theory to classify all stationary points as either saddle points (transition states, with a single imaginary frequency) or energy minima (reactants, intermediates and products, with only real frequencies). These calculations were also used to obtain the thermochemistry corrections (zero-point, thermal and entropy energies) at the experimental p = 1 atm and T = 298.15 K. All

optimizations needed for the proposed mechanism were carried out in solvent (dichloromethane) with the SMD solvation model.[17]

Optimized coordinates and energies including solvation (DCM)

3

```
E = -770.3875419
G = -770.17192
 Au                 0.67014800   -0.80671000   -0.00031600
 N                  1.08341700    1.25117300   -0.00624100
 C                  0.44508500   -2.88115700    0.11278500
 H                  0.33634200   -3.08505900    1.17681500
 H                 -0.30478500   -3.33318200   -0.52729200
 C                 -0.01698100    2.03958000   -0.01137900
 C                 -1.29101500    1.31762300   -0.01091500
 C                  2.31377000    1.77691300    0.00142800
 H                  3.13761600    1.07404200    0.00702100
 C                 -1.22909000   -0.08773300    0.00556100
 C                 -2.39941600   -0.83111600    0.01035600
 H                 -2.35952400   -1.91601400    0.01812200
 C                 -3.65292400   -0.20578100    0.00075000
 C                  0.13316900    3.42418400   -0.01124400
 H                 -0.73975700    4.06423100   -0.01337900
 C                 -3.70010500    1.18938100   -0.02275800
 H                 -4.66296400    1.69079600   -0.03984800
 C                  2.51727500    3.14329100    0.00229500
 H                  3.52551100    3.53799600    0.00882500
 C                 -2.53750200    1.94596400   -0.02754900
 H                 -2.61363600    3.02799800   -0.04945100
 C                  1.40353000    3.97590600   -0.00471600
 H                  1.52492900    5.05361400   -0.00403100
 C                 -4.90923500   -1.02007200    0.03769900
 H                 -5.19242000   -1.24184200    1.07333700
 H                 -4.77839400   -1.97595100   -0.47582000
 H                 -5.74328500   -0.48614200   -0.42379900
 C                  1.76661800   -2.69480800   -0.42327200
 H                  1.93563700   -2.88084400   -1.48115400
 C                  2.68535600   -1.93915700    0.28074800
 H                  2.64418500   -1.87456700    1.36615000
 H                  3.59371200   -1.60129700   -0.20556400
```

TS3-4

```
E = -770.3653204
G = -770.150245
 Au                -0.25937100   -1.00551900   -0.26493000
 N                  1.79452100   -0.50924800    0.02238400
 C                 -2.21854700   -1.52181700   -0.54791400
 H                 -2.88877300   -0.66843800   -0.60770900
 H                 -2.16472000   -2.01947000   -1.52447700
 C                  1.99858100    0.81685800    0.15198800
 C                  0.78047600    1.62676000    0.09247500
```

C	2.81058300	-1.37291900	0.06807400
H	2.55991900	-2.42245500	-0.04161500
C	-0.45717800	0.98705500	-0.10650200
C	-1.63972900	1.69193800	-0.16049900
H	-2.59056300	1.19720000	-0.30616800
C	-1.63151800	3.08868400	-0.02391200
C	3.28893000	1.30583400	0.33168100
H	3.46315300	2.36912200	0.43289100
C	-0.41186100	3.73220200	0.16979200
H	-0.38831200	4.81184000	0.27718200
C	4.11444700	-0.94826000	0.24784300
H	4.91933600	-1.67210000	0.28241300
C	0.77421400	3.01561500	0.22794900
H	1.70697700	3.54799300	0.38195000
C	4.35013600	0.41567400	0.37951900
H	5.35980300	0.78609200	0.51983300
C	-2.92128900	3.84706000	-0.07534300
H	-3.55391800	3.59462900	0.78238000
H	-3.48722200	3.59868900	-0.97833800
H	-2.74633900	4.92448300	-0.06307000
C	-2.56767600	-2.45401200	0.54192100
C	-3.38173200	-2.15401400	1.56021000
H	-2.12573700	-3.44921600	0.50001400
H	-3.60482500	-2.87864800	2.33708500
H	-3.85716000	-1.17892900	1.63671100

4
E = -770.3656924
G = -770.15237

Au	-0.01302100	-1.13377400	-0.14054300
N	1.93435900	-0.27191500	0.04791000
C	-1.83688900	-2.00776600	-0.38089800
H	-2.28182500	-1.68594500	-1.32471500
H	-1.43725800	-3.03296500	-0.49015100
C	1.91266300	1.07613200	0.08197000
C	0.57149500	1.65826300	0.00986900
C	3.08331500	-0.94713600	0.10902400
H	3.01248200	-2.02876400	0.07442400
C	-0.53513200	0.79691600	-0.08167300
C	-1.82838400	1.25594600	-0.12194400
H	-2.66777100	0.57011800	-0.16174500
C	-2.07891900	2.63617100	-0.07951800
C	3.10470500	1.78685600	0.18490800
H	3.09845000	2.86915700	0.21341600
C	-0.99300200	3.50550200	0.00291800
H	-1.16923800	4.57571800	0.03944700
C	4.29999300	-0.29731900	0.21208300
H	5.21749000	-0.87043000	0.26085800
C	0.30931400	3.02839800	0.04901700
H	1.12879400	3.73503200	0.12470800
C	4.30276600	1.09246900	0.25028400
H	5.23780400	1.63604500	0.33112500
C	-3.48724300	3.13957200	-0.12940700
H	-4.11427800	2.62540500	0.60516900

H	-3.92940000	2.95905300	-1.11511900
H	-3.52957400	4.21234100	0.06855700
C	-2.76819700	-1.91357600	0.75238200
C	-3.99341200	-1.38517900	0.66951500
H	-2.42462800	-2.30536600	1.70836300
H	-4.64758300	-1.33341100	1.53444600
H	-4.38373300	-1.00595400	-0.27241100

TS4-5
E = -770.3572465
G = -770.1437

Au	-0.19272900	-1.05341000	-0.21689200
N	1.85626200	-0.52257900	0.01166900
C	-2.15087900	-1.54773200	-0.43664500
H	-2.54028600	-0.93832600	-1.25835500
H	-2.10200300	-2.59131100	-0.77995900
C	2.03827800	0.80627500	0.15401700
C	0.80580800	1.59337600	0.09405900
C	2.88743600	-1.36823800	0.04706900
H	2.65512900	-2.42103200	-0.07153900
C	-0.41726500	0.92611900	-0.09520200
C	-1.61624600	1.59690000	-0.18845300
H	-2.55074700	1.07273000	-0.34544500
C	-1.63960300	2.99640800	-0.08451700
C	3.31923700	1.31663200	0.33718300
H	3.47338500	2.38195600	0.45055700
C	-0.43697900	3.66978200	0.11821300
H	-0.43966100	4.75152600	0.20472100
C	4.18405800	-0.92178600	0.22768300
H	5.00168400	-1.63154800	0.25262000
C	0.76559800	2.98364500	0.20391400
H	1.68431500	3.54023900	0.35649700
C	4.39609100	0.44444500	0.37355300
H	5.39938700	0.83077500	0.51645300
C	-2.94053400	3.72733200	-0.20283800
H	-3.67419800	3.34064500	0.51092300
H	-3.36622200	3.60051100	-1.20381000
H	-2.81137900	4.79592800	-0.02074400
C	-2.98275700	-1.40425400	0.79153200
C	-4.01352800	-2.19447000	1.08965500
H	-2.79277300	-0.53677100	1.42197800
H	-4.63086200	-2.00478400	1.96283700
H	-4.26630100	-3.05972300	0.48072100

5
E = -770.3662614
G = -770.152925

Au	-0.24139900	-1.01123900	-0.27132400
N	1.80658800	-0.48924000	0.01566700
C	-2.19177300	-1.52546400	-0.54634900
H	-2.67199500	-0.82539800	-1.23050800
H	-2.05996600	-2.49803100	-1.04439100
C	1.98845200	0.84053100	0.15059000
C	0.75681400	1.63123400	0.09766700

C	2.83813800	-1.33448700	0.05270300
H	2.60583400	-2.38771900	-0.06134000
C	-0.46713600	0.96907200	-0.10408800
C	-1.66828400	1.64231000	-0.15061600
H	-2.60520200	1.12030000	-0.30195900
C	-1.69101300	3.03761600	-0.00143400
C	3.27049700	1.35082500	0.32821300
H	3.42558600	2.41656400	0.43425400
C	-0.48467200	3.70600200	0.19608600
H	-0.48619500	4.78468200	0.31511200
C	4.13516100	-0.88780000	0.22933900
H	4.95262400	-1.59769200	0.25666100
C	0.71926400	3.01824600	0.24509900
H	1.63840500	3.57240200	0.40397200
C	4.34753700	0.47919600	0.36707100
H	5.35114200	0.86658400	0.50492200
C	-2.99512400	3.77054500	-0.05822400
H	-3.69577700	3.38173600	0.68730300
H	-3.46766900	3.64896700	-1.03846900
H	-2.85675600	4.83803800	0.12373200
C	-2.87307300	-1.65737000	0.75407800
C	-3.05163900	-2.81623800	1.39616400
H	-3.23910400	-0.73756900	1.20880600
H	-3.55441900	-2.85768300	2.35739000
H	-2.71097000	-3.75793900	0.97162300

TS5-3'

E = -770.3631154

G = -770.148732

Au	-0.43075300	-0.91832400	-0.22144400
N	1.67804300	-0.75169200	0.07297800
C	-2.42773700	-1.10539800	-0.58092000
H	-2.85043600	-0.19903100	-1.00972700
H	-2.42455900	-1.90829200	-1.33133000
C	2.09281000	0.53147700	0.10258400
C	1.01417100	1.52067500	0.05769000
C	2.55060700	-1.76011800	0.12027300
H	2.13579500	-2.76170800	0.09052200
C	-0.31265200	1.07709400	-0.08430600
C	-1.37965600	1.94937200	-0.07626600
H	-2.39966400	1.59814800	-0.16825600
C	-1.15384300	3.32815200	0.05917500
C	3.45196100	0.82142400	0.17513100
H	3.79132300	1.84958700	0.18617300
C	0.15812600	3.78012300	0.18234700
H	0.34805600	4.84355600	0.28608700
C	3.91293600	-1.53599200	0.20176300
H	4.59805400	-2.37384700	0.24017200
C	1.22488500	2.89351500	0.18582100
H	2.23408300	3.27364000	0.30209800
C	4.36413600	-0.22097400	0.22472200
H	5.42609700	-0.00703200	0.27845000
C	-2.31325400	4.27520600	0.07027700
H	-2.87642600	4.21306600	-0.86638900

H	-1.97859300	5.30611400	0.20112600
H	-3.00659100	4.03297700	0.88182800
C	-3.04372700	-1.52561200	0.68860500
C	-3.25482600	-2.80207300	1.03246900
H	-3.31513400	-0.73662000	1.38859700
H	-3.69281000	-3.06382900	1.99065700
H	-3.00960700	-3.61854100	0.35704600

TS (Cs)
E = -770.3569017
G = -770.143445

Au	0.67448900	-0.69548700	0.00185600
N	-1.38096700	-1.24246800	-0.00137800
C	2.62403100	-0.11342700	0.00429700
H	2.79917900	0.50447800	-0.88076600
H	2.79947000	0.49448700	0.89631000
C	-2.20299500	-0.17229200	0.00066800
C	-1.51231500	1.11964300	0.00090600
C	-1.86465700	-2.48582700	-0.00319800
H	-1.13588700	-3.28916900	-0.00484800
C	-0.10682600	1.13780100	0.00203800
C	0.62053100	2.30657700	-0.00143400
H	1.70446300	2.29769400	-0.00227800
C	-0.05004300	3.53925200	-0.00524200
C	-3.58201000	-0.35785200	0.00226100
H	-4.25182200	0.49234000	0.00540300
C	-1.44354100	3.53880700	-0.00681300
H	-1.97600800	4.48438500	-0.01260600
C	-3.22515700	-2.73396100	-0.00302900
H	-3.58912800	-3.75376400	-0.00496100
C	-2.16426600	2.35366800	-0.00341200
H	-3.24807800	2.39591100	-0.00712000
C	-4.09180700	-1.64714800	0.00015500
H	-5.16498400	-1.80329100	0.00101800
C	0.73146600	4.81590900	0.00257500
H	1.23237400	4.95757000	0.96634700
H	0.08139200	5.67567600	-0.17086000
H	1.50841500	4.80679900	-0.76734300
C	3.46462100	-1.35718800	-0.00372700
C	4.79558000	-1.36917000	-0.00761700
H	2.94648200	-2.32001900	-0.00754900
H	5.34813200	-2.30387500	-0.01378700
H	5.37249700	-0.44720100	-0.00451500

3-NTf_2
E = -2596.791091
G = -2596.542798

Au	-1.64827400	-0.50311700	-0.93689700
N	-1.30912300	1.50448900	-0.41578000
C	-3.44254900	-0.07475000	-0.08204300
C	-4.71858900	1.68796400	0.97706400
H	-4.80862400	2.70683900	1.33954600
C	-0.16841400	2.16641100	-0.63884400
H	0.62591200	1.61707400	-1.12778700

C	-4.52791000	-0.92935300	0.05927200
H	-4.46680600	-1.95467900	-0.29197500
C	-5.79447200	0.82457300	1.10618100
H	-6.71156900	1.17831900	1.56813600
C	-1.84346600	-2.46887000	-1.61208600
H	-2.28868100	-3.18407900	-0.92882100
H	-2.33648700	-2.37791400	-2.57861000
C	-3.53247600	1.24777400	0.38430800
C	-5.71831000	-0.49554100	0.65261300
C	-2.35179200	2.09678600	0.21171800
C	-2.23634300	3.41656600	0.63844800
H	-3.06408900	3.89361900	1.14763700
C	-0.00363900	3.47902600	-0.23776800
H	0.93551300	3.98366900	-0.42860700
C	-1.05814600	4.10960600	0.41143500
H	-0.96268700	5.13781300	0.74369300
C	-0.42342000	-2.25512200	-1.53338000
H	0.13693600	-2.69507600	-0.71270900
C	-6.89060500	-1.41793100	0.79032400
H	-6.63267800	-2.43667400	0.49269300
H	-7.72488800	-1.08322700	0.16425600
H	-7.25275300	-1.44269800	1.82276800
C	0.14941800	-1.20712200	-2.23006700
H	-0.29185300	-0.84504600	-3.15699300
H	1.15458800	-0.88467800	-1.98125300
S	3.65370600	0.89372000	-0.13603700
S	2.74618600	-1.33393300	1.37815100
F	0.58663700	0.17429700	1.57767000
F	5.45564000	0.88026000	-2.04442000
F	5.10950700	-1.05577900	-1.14914100
F	1.55936800	-0.22547000	3.46354300
F	0.41174400	-1.77557100	2.48950800
O	2.22822500	-1.94473900	0.16254000
O	3.43707900	-2.17064400	2.34159900
O	2.63495700	0.65227500	-1.15054000
N	3.55317500	0.03150900	1.19961800
F	6.25099100	0.43785100	-0.08542900
O	3.95845000	2.26713100	0.22136600
C	1.23092600	-0.74533300	2.28942900
C	5.22320500	0.24397200	-0.90044900

4-NTf$_2$
E = -2596.798744
G = -2596.548646

Au	-0.67221800	-0.27748500	0.05195800
N	-1.02497000	1.77516400	-0.52536600
C	-0.54747400	-2.23935800	0.67711900
H	-1.12860700	-2.80117100	-0.05875900
H	0.50260500	-2.52252600	0.59174500
C	-2.33493000	2.09982100	-0.47806200
C	-3.24145400	0.97905500	-0.22116500
C	-0.10955400	2.69743400	-0.82445200
H	0.91695300	2.36518200	-0.87389800
C	-2.68259500	-0.29259700	0.01154000

C	-3.51913400	-1.39020800	0.15351700
H	-3.11213000	-2.38434000	0.29491200
C	-4.91033700	-1.25858500	0.11050400
C	-2.73481100	3.41549100	-0.70170600
H	-3.78216400	3.68270500	-0.64665400
C	-5.45317200	0.01174700	-0.09117400
H	-6.53119200	0.13627600	-0.12862200
C	-0.44498400	4.01540700	-1.07279100
H	0.33007900	4.73192000	-1.31501600
C	-4.63226900	1.11241300	-0.26348600
H	-5.08287400	2.08211900	-0.44659000
C	-1.78381600	4.37832100	-0.99584900
H	-2.08721100	5.40505800	-1.17051500
C	-5.79831300	-2.45189300	0.28818800
H	-6.31944500	-2.40570000	1.25094800
H	-5.22642900	-3.38191000	0.25982600
H	-6.56473400	-2.49201200	-0.49154300
C	-1.03679200	-2.43779200	2.05974800
C	-0.26723300	-2.82757300	3.07694000
H	-2.09620200	-2.26999900	2.24260000
H	-0.68262400	-2.98041800	4.06895600
H	0.79848000	-2.99618600	2.95206800
S	2.43198900	-0.05372100	1.32326900
S	2.18934600	-0.22693100	-1.54119300
F	1.16881100	-2.61830100	-1.99761800
F	3.52876300	1.89963100	2.64267200
F	1.62634900	2.41548200	1.76022900
F	2.99673500	-2.65295200	-0.84807500
F	3.05883700	-2.22653900	-2.96462100
O	1.17767100	0.20786700	-2.48288600
O	3.53167700	0.30514000	-1.60015600
O	1.56735900	-0.39582700	2.42953600
N	1.51396600	-0.11524500	-0.04514400
F	3.39390000	2.29179000	0.52420700
O	3.72861100	-0.67352200	1.16878500
C	2.36301400	-2.06705400	-1.84750700
C	2.76773100	1.77070600	1.56410900

5-NTf$_2$
E = -2596.798698
G = -2596.548535

Au	0.70131700	-0.19103200	-0.09833200
N	0.97452500	1.91444500	0.24011900
C	0.61513000	-2.22010300	-0.48733900
H	1.22369500	-2.74348600	0.25270200
H	-0.43507800	-2.46611800	-0.31060800
C	2.27591500	2.26731500	0.30653200
C	3.22276300	1.16039800	0.16987400
C	0.01352600	2.83420900	0.33616700
H	-1.00359500	2.47820300	0.26477500
C	2.70782300	-0.13478500	-0.04935500
C	3.57397400	-1.20424000	-0.16945800
H	3.19466000	-2.20302200	-0.34487600
C	4.96162800	-1.03388600	-0.08345700

C	2.62571300	3.60359500	0.48691700
H	3.66814800	3.89093500	0.53823000
C	5.46200300	0.24959900	0.12590700
H	6.53443700	0.40418500	0.19303600
C	0.29784400	4.17507900	0.51647500
H	-0.51038400	4.89217800	0.59088900
C	4.60480000	1.33272300	0.25181100
H	5.02253600	2.31998900	0.41743600
C	1.63056000	4.56080300	0.59487300
H	1.89461700	5.60357600	0.73517500
C	5.86951200	-2.21836700	-0.20848000
H	5.65933400	-2.77945700	-1.12460600
H	5.72753600	-2.90842600	0.63018300
H	6.91869700	-1.91570400	-0.22364300
C	0.98759700	-2.56137100	-1.87846000
C	1.91738500	-3.45606600	-2.22041200
H	0.42425200	-2.05963700	-2.66235300
H	2.12246300	-3.68277700	-3.26270900
H	2.48715300	-4.00520900	-1.47416100
S	-2.37284000	-0.43000100	-1.33604700
S	-2.20556400	-0.01422700	1.49492300
F	-0.80281700	-2.04767300	2.41913900
F	-3.54207700	1.25205000	-2.95280100
F	-2.91403400	2.17151800	-1.10446900
F	-2.64200800	-2.62961900	1.44583600
F	-2.69721000	-1.71345700	3.40002000
O	-1.35239900	0.79500700	2.33922000
O	-3.62394600	0.25681000	1.38284400
O	-1.40997900	-0.40986200	-2.41832800
N	-1.49504100	-0.09352400	0.02047400
F	-4.66817400	0.91220600	-1.15090200
O	-3.29212100	-1.53542500	-1.17999000
C	-2.07473200	-1.73216000	2.22899800
C	-3.45977700	1.08893800	-1.64038900

TS4-5-NTf$_2$
E = -2596.79363
G = -2596.544097

Au	-0.64410400	-0.24368600	-0.01783100
N	-0.98471600	1.83629100	-0.45662500
C	-0.54070300	-2.24172000	0.42350500
H	-1.09181800	-2.75975300	-0.36673900
H	0.51085900	-2.51832300	0.32493800
C	-2.29929800	2.14692900	-0.48106400
C	-3.20795200	1.01791100	-0.27275200
C	-0.06190100	2.77923000	-0.65296600
H	0.97025800	2.46206100	-0.62992900
C	-2.64878700	-0.25320200	-0.03949800
C	-3.48214200	-1.34773200	0.13982200
H	-3.07094800	-2.33455500	0.31547400
C	-4.87337300	-1.21620200	0.10491400
C	-2.69941500	3.46328900	-0.69955100
H	-3.75137700	3.71721400	-0.71125700
C	-5.41773600	0.04929400	-0.12389500

H	-6.49608100	0.17230300	-0.15854000
C	-0.39680200	4.10087200	-0.88132900
H	0.38397600	4.83503300	-1.03675100
C	-4.59922200	1.14931500	-0.31227000
H	-5.05221200	2.11777200	-0.49606500
C	-1.74275500	4.44461700	-0.89900400
H	-2.04680000	5.47195400	-1.06895000
C	-5.75975600	-2.40542500	0.31586300
H	-6.32537000	-2.30679400	1.24882600
H	-5.17975100	-3.32919600	0.36927900
H	-6.48897100	-2.50157800	-0.49451200
C	-1.04482500	-2.61031500	1.79007400
C	-0.97126300	-3.84385800	2.28503200
H	-1.48321600	-1.82704300	2.40363100
H	-1.33890100	-4.07176300	3.28141800
H	-0.53582300	-4.66388800	1.71733700
S	2.34409600	-0.21522700	1.41344500
S	2.32375400	-0.03740000	-1.46598600
F	1.28387600	-2.33124600	-2.24832100
F	3.29507400	1.58451200	3.04024200
F	1.43594800	2.15737300	2.10067800
F	3.05109100	-2.54362500	-1.02465200
F	3.22737300	-1.87843600	-3.07243400
O	1.39400500	0.53582700	-2.41755000
O	3.67624000	0.45985000	-1.36097900
O	1.40111800	-0.71780300	2.38855000
N	1.54048000	-0.09524200	-0.02074000
F	3.28212700	2.22498900	0.97883300
O	3.66315300	-0.79102700	1.28679400
C	2.47980700	-1.83097900	-1.97902800
C	2.60761200	1.56890300	1.90699000

3-Pt

E = -754.311932187

G = -754.097069

Pt	0.69104200	-0.82142200	0.00087600
N	1.06522500	1.22654400	-0.01050600
C	0.46591700	-2.89344000	0.16835500
H	0.40972800	-3.13280100	1.23021600
H	-0.26391600	-3.41354200	-0.44607200
C	-0.03221500	2.02422700	-0.00211400
C	-1.30262900	1.30043900	0.00290000
C	2.28860000	1.77123700	-0.01670900
H	3.11733200	1.07496300	-0.02426300
C	-1.20086100	-0.10927100	-0.00474500
C	-2.39743500	-0.83349300	-0.01148100
H	-2.36365200	-1.91973000	-0.02303500
C	-3.65048800	-0.21565100	-0.00753700
C	0.11272700	3.41063800	-0.00239300
H	-0.76608200	4.04304000	0.00196700
C	-3.71005800	1.18199300	0.00198800
H	-4.67565800	1.67954400	0.00333600
C	2.49105100	3.13866400	-0.01434300
H	3.49946000	3.53322500	-0.01837200

C	-2.54759500	1.93490400	0.00716200
H	-2.61943500	3.01826000	0.01183600
C	1.37800900	3.97128100	-0.00763500
H	1.49587400	5.04945500	-0.00676300
C	-4.91130600	-1.02809600	0.00077400
H	-5.42258200	-0.94831200	0.96680400
H	-4.70432700	-2.08504800	-0.18137900
H	-5.61374400	-0.67924700	-0.76249000
C	1.74352500	-2.61822000	-0.42010300
H	1.88396700	-2.80224800	-1.48322200
C	2.66660000	-1.80553100	0.25283300
H	2.75191200	-1.83993700	1.33695600
H	3.55019400	-1.47056800	-0.28197200

References

[1] E. Langseth, C. H. Görbitz, R. H. Heyn and M. Tilset, *Organometallics* **2012**, *31*, 6567-6571.

[2] V. Dyadkin, P. Pattison, V. Dmitriev and D. Chernyshov, *J. Synchrotron Rad.* **2016**, *23*, 825-829.

[3] Rigaku Oxford Diffraction **2015**, *CrysAlis PRO 1.171.38.41*. Rigaku OD, Yarnton, England.

[4] G. Sheldrick, *Acta Crystallogr. Sect. A* **2015,** *71* (1), 3-8.

[5] G. Sheldrick, *Acta Crystallogr. Sect. C* **2015,** *71* (1), 3-8.

[6] O. V. Dolomanov, L. J. Bourhis, R. J. Gildea, J. A. K. Howard, and H. J. Puschmann, *J. Appl. Crystallogr.* **2009,** *42* (2), 339-341.

[7] F. H. Allen, O. Johnson, G. P. Shields, B. R. Smith, and M. J. Towler, *J. Appl. Crystallogr.* **2004,** *37* (2), 335-338.

[8] H. Putz and K. Brandenburg, K. *Diamond - Crystal and Molecular Structure Visualization, Ver. 4.1.2*, Crystal Impact: Kreuzherrenstr. 102, 53227 Bonn, Germany, **1997**.

[9] *Bruker APEX3, SAINT, SADABS, XPREP Ver. 2016.5-0*, Bruker AXS inc, Madison, Wisconsin, USA, **2016**.

[10] A. Spek, *J. Appl. Crystallogr.* **2003,** *36* (1), 7-13.

[11] M. J. Frisch, G. W. Trucks, H. B. Schlegel, G. E. Scuseria, M. A. Robb, J. R. Cheeseman, G. Scalmani, V. Barone, B. Mennucci, G. A. Petersson, H. Nakatsuji, M. Caricato, X. Li, H. P. Hratchian, A. F. Izmaylov, J. Bloino, G. Zheng, J. L. Sonnenberg, M. Hada, M. Ehara, K. Toyota, R. Fukuda, J. Hasegawa, M. Ishida, T. Nakajima, Y. Honda, O. Kitao, H. Nakai, T. Vreven, J. J. A. Montgomery, J. E. Peralta, F. Ogliaro, M. Bearpark, J. J. Heyd, E. Brothers, K. N. Kudin, V. N. Staroverov, R. Kobayashi, J. Normand, K. Raghavachari, A. Rendell, J. C. Burant, S. S. Iyengar, J. Tomasi, M. Cossi, N. Rega, J. M. Millam, M. Klene, J. E. Knox, J. B. Cross, V. Bakken, C. Adamo, J. Jaramillo, R. Gomperts, R. E. Stratmann, O. Yazyev, A. J. Austin, R. Cammi, C. Pomelli, J. W. Ochterski, R. L. Martin, K. Morokuma, V. G. Zakrzewski, G. A. Voth, P. Salvador, J. J. Dannenberg, S. Dapprich, A. D. Daniels, Ö. Farkas, J. B. Foresman, J. V. Ortiz, J. Cioslowski and D. J. Fox, *Gaussian 09, Revision D.01*, Gaussian, Inc., Wallingford CT, 2009.

[12] C. Adamo and V. J. Barone, *J. Chem. Phys.*, **1999**, *110*, 6158-6170.

[13] S. Grimme, J. Antony, S. Ehrlich and H. Krieg, *J. Chem. Phys.*, **2010**, *132*, 154104-154123.

[14] a) M. S. M. Holmsen, A. Nova, D. Balcells, E. Langseth, S. Øien-Ødegaard, R. H. Heyn, M. Tilset and G. Laurenczy, *ACS Catal.*, **2017**, *7*, 5023-5034. b) E. Langseth, M. L. Scheuermann, D. Balcells, W. Kaminsky, K. I. Goldberg, O. Eisenstein, R. H. Heyn and M. Tilset, *Angew.*

Chem. Int. Ed., **2013**, *52*, 1660-1663. C) E. Langseth, A. Nova, E. A. Tråseth, F. Rise, S. Øien, R. H. Heyn and M. Tilset, *J. Am. Chem. Soc.*, **2014**, *136*, 10104-10115. d) D. Balcells, O. Eisenstein, M. Tilset and A. Nova, *Dalton Trans.*, **2016**, *45*, 5504-5513. e) M. S. M. Holmsen, A. Nova, D. Balcells, E. Langseth, S. Øien-Ødegaard, E. A. Traseth, R. H. Heyn and M. Tilset, *Dalton Trans.*, **2016**, *45*, 14719-14724.

[15] a) R. Krishnan, J. S. Binkley, R. Seeger and J. A. Pople, *J. Chem. Phys.*, **1980**, *72*, 650-654. b) A. D. McLean and G. S. Chandler, *J. Chem. Phys.*, **1980**, *72*, 5639-5648.

[16] a) D. Figgen, K. A. Peterson, M. Dolg and H. Stoll, *J. Chem. Phys.*, **2009**, *130*, 164108-164120. b) D. Figgen, G. Rauhut, M. Dolg and H. Stoll, *Chem. Phys.*, **2005**, *311*, 227-244.

[17] A. V. Marenich, C. J. Cramer and D. G. Truhlar, *J. Phys. Chem. B*, **2009**, *113*, 6378-6396.
